# Assessing the Gene Content of the Megagenome: Sugar Pine (*Pinus lambertiana*)

**DOI:** 10.1534/g3.116.032805

**Published:** 2016-10-31

**Authors:** Daniel Gonzalez-Ibeas, Pedro J. Martinez-Garcia, Randi A. Famula, Annette Delfino-Mix, Kristian A. Stevens, Carol A. Loopstra, Charles H. Langley, David B. Neale, Jill L. Wegrzyn

**Affiliations:** * Department of Ecology and Evolutionary Biology, University of Connecticut, Storrs, Connecticut 06269; † Department of Plant Sciences, University of California, Davis, California 95616; ‡ United States Department of Agriculture Forest Service, Institute of Forest Genetics, Placerville, California 95667; § Department of Evolution and Ecology, University of California, Davis, California 95616; ** Department of Ecosystem Science and Management, Texas A&M University, College Station, Texas 77843

**Keywords:** conifer, transcriptome, dicer, gymnosperm, DCL, RNA-Seq

## Abstract

Sugar pine (*Pinus lambertiana* Douglas) is within the subgenus Strobus with an estimated genome size of 31 Gbp. Transcriptomic resources are of particular interest in conifers due to the challenges presented in their megagenomes for gene identification. In this study, we present the first comprehensive survey of the *P. lambertiana* transcriptome through deep sequencing of a variety of tissue types to generate more than 2.5 billion short reads. Third generation, long reads generated through PacBio Iso-Seq have been included for the first time in conifers to combat the challenges associated with *de novo* transcriptome assembly. A technology comparison is provided here to contribute to the otherwise scarce comparisons of second and third generation transcriptome sequencing approaches in plant species. In addition, the transcriptome reference was essential for gene model identification and quality assessment in the parallel project responsible for sequencing and assembly of the entire genome. In this study, the transcriptomic data were also used to address questions surrounding lineage-specific Dicer-like proteins in conifers. These proteins play a role in the control of transposable element proliferation and the related genome expansion in conifers.

Gymnosperm genomes are among the largest sequenced to date. Their 14-fold variation between the minimum (*Gnetum ula*: 4.54 pg) and maximum (*Pinus gerardiana*: 57.35 pg) is much lower than the 1000-fold variation seen in angiosperms (1C = 0.05 ± 127.4 pg) (Leitch *et al.* 2001). Interestingly, estimates of the total number of genes seems relatively constant across all land plants, ranging from 25,000 to 45,000, as observed recently in Norway spruce (Nystedt *et al.* 2013) as well as smaller genomes such as *Arabidopsis thaliana* (Swarbreck *et al.* 2008) or *Gossypium arboreum* (Li *et al.* 2014a). The cone bearing gymnosperms belonging to the Pinales order inhabit some of the largest ecosystems on earth, contributing significantly to global carbon assimilation. Within the Pinales, the Pinaceae are the largest extant conifer family with over 200 species. Their genomes have remarkable characteristics, including a constant number of chromosomes, enormous size, and a high proportion of repetitive elements (Neale *et al.* 2014; Nystedt *et al.* 2013). Despite challenges, inexpensive next-generation sequencing and custom assembly approaches produced two draft pine genomes (*P. taeda* and *P. lambertiana*) at 22 and 31 Gbp, respectively (Neale *et al.* 2014; Stevens *et al.*, 2016).

*P. lambertiana* is a member of the genus *Pinus*, and is within the subgenus Strobus, which includes members known collectively as the white pines or five-needle pines. *P. lambertiana* occupies a variety of habitats throughout the Cascade range in Oregon to as far south as Baja California, Mexico. The majority of its range occurs in the mixed conifer forests of the Sierra Nevada (Kinloch and Scheuner 1990). This tall and voluminous species shares habitat with several other tree species, and is rarely found in pure stands (Fites-Kaufman *et al.* 2007). Disturbances such as historical logging, climate change, and introduction of the nonnative pathogen *Cronartium ribicola*, have sharply reduced *P. lambertiana* populations (Maloney *et al.* 2011).

The conifer genomes have already contributed to advancements in conifer biology (Li *et al.* 2015); however, the fragmented nature of the final assemblies (each containing over 14 million scaffolds) supports the need for comprehensive transcriptomic resources (Visser *et al.* 2015). Recent advancements in transcriptome characterization, through techniques such as RNA-seq, have contributed to improved resolution of transcripts, and the subsequent ability to quantify gene expression in thousands of genes at a time (Conesa *et al.* 2016; Kanitz *et al.* 2015). Short read technologies, available through the numerous Illumina platforms, provide substantial depth at a low cost with reads that typically range from 50 to 300 nucleotides (nt) in length (Cahill *et al.* 2010). In the absence of a contiguous genome assembly, researchers rely on *de novo* assembly techniques to organize those short reads into full-length transcripts (Moreton *et al.* 2015). Recently, the precision and sensitivity of RNA-seq have come into question, especially for transcriptome reconstruction (Korf 2013). A relatively new method known as “Isoform Sequencing” (Iso-Seq), developed by Pacific Biosciences (PacBio), is capable of identifying new isoforms up to 6 kb in length due to its long read, single molecule sequencing technology. This methodology has been used independently, as well as in combination with short read approaches to improve transcript identification. The Iso-Seq approach has been applied to human tumor cell lines and recently to select plant genomes (Dong *et al.* 2015; Xu *et al.* 2015). To date, the effectiveness of long read transcriptome sequencing approaches has been evaluated shallowly in select angiosperms and never in conifers.

Extensive transcriptome resources have been developed for several conifer species, particularly those of tremendous economic value. Early work has included cDNA microarrays to examine expression responses to biotic and abiotic stressors ranging from 1248 (Myburg *et al.* 2006) to 26,496 ESTs (Lorenz *et al.* 2011b). Following this, large-scale Sanger-based EST sequencing produced hundreds of thousands of sequences with the greatest contributions to *P. taeda* and *Picea glauca*, both having over 300,000 sequences in GenBank (Mackay and Dean 2011). Among pines within the subgenus *Strobus*, very few resources have been developed. In this study, we have implemented PacBio Iso-Seq for the first time in conifers to improve the accuracy of transcript construction and evaluate its utility against traditional, short read, deep sequencing approaches. Novel sequencing approaches combined with comprehensive tissue sampling provides the greatest depth and most detailed analysis of a white pine transcriptome to date.

The recent availability of a draft *P. lambertiana* genome sequence, coupled with transcriptomics, offers opportunities to study basic questions about the biology of conifers as it relates to genome evolution and gene expression. Genome sequencing in conifers has led to observations of genome expansion resulting primarily from transposable element (TE) proliferation rather than genome duplications (Wegrzyn *et al.* 2014; Nystedt *et al.* 2013). The peculiar profile of the small RNAs population in these plant species, and the previous identification of potential lineage-specific, Dicer-like (DCL) proteins (Dolgosheina *et al.* 2008), raises questions about whether the mechanism for controlling genome size through epigenetic modifications works differently in gymnosperms. In this study, we take advantage of the characterized transcriptome to provide new insight on conventional and conifer-specific DCLs.

## Materials and Methods

### Plant material

A comprehensive collection of tissues was made from 12 existing *P. lambertiana* trees (11-91 6000, 11-92 6000, 11-94 6000, 11-99 5701, JJ-86 11,101, JJ-101 11,105, GG 79 15,306, V-120 18,856, E-109 7392, B-109 BLM-8, JJ-105 11,200, 11-105 5503) in the clone bank at Badger Hill in the El Dorado National Forest in California (USDA Forest Service). This collection included: megagametophytes; embryos; cotyledon stage seedlings before development of primary needles, containing only cotyledons, stem, and root (labeled “basket” stage); primary needle stage seedlings; pollen; early female cones before pollination; female cones near pollination; 2 cm female cones after pollination; stems; and roots. From the same clone bank, open pollinated seeds were collected. Seeds were germinated and established seedlings were used for various treatments conducted at the Institute for Forest Genetics (Placerville, CA). Two grown seedlings were used to simulate a salt stress via a soil drench using large quantities of 200 mM NaCl, before harvesting all three tissues after 2 hr. To study effects of wounding, needle nose pliers were used to crush needles and stems while still on the tree. We harvested needles and stems after 4 hours. To simulate pathogen or insect attack, trees were treated with Jasmonic acid (JA) [100 μm JA plus 0.02% tween (a wetting agent)]. This solution was applied as a drench to the roots and sprayed on the foliage. Needles, stems, and roots were harvested after 4 hr of inoculation, but only the stem was used in our analysis. Tissues from samples were separately harvested in needles, roots, and stems and collected in 50 ml tubes. In order to preserve the integrity of the drought stress treatment, samples were frozen immediately, as water could initiate reversal.

### Library construction and sequencing

Total RNA was isolated by adapting the method described by Sangha *et al.* (2010), which combined a CTAB-based lysis solution with the silica column-based RNA binding, DNase, and washing steps from an RNeasy Plant Mini Kit (Qiagen, Germany). RNA quality was evaluated using the Agilent Bioanalyzer 2100 (Agilent Technologies, Folsom, CA). All Illumina libraries were constructed at the Vincent J. Coates Genome Sequencing Laboratory (University of California, Berkeley) on the IntegenX Apollo 324 robot (Wafergen, Fremont, CA). Illumina MiSeq libraries were constructed with an insert of 500 nt, and sequenced in individual lanes, 300 nt PE, 600 cycles, using Version 3 chemistry (Illumina, San Diego, CA). RNA samples for the Illumina HiSeq were treated prior to library construction with a Ribo-Zero rRNA Removal Kit (Plant) (Illumina). Nine HiSeq 2000 libraries were constructed with standard insert sizes, and sequenced as 100 nt PE in individual lanes (Illumina). PacBio Iso-Seq libraries were constructed following the PacBio modified protocol using the Clontech SMARTer PCR cDNA Synthesis Kit and Blue Pippin Size Selection System. Insert sizes were selected for the following inserts: 1, 2, and 3–6 kb (Sage Science, Beverly, MA). Libraries were then prepared using the SMRTbell library protocol (PacBio, Menlo Park, CA). Each library was sequenced across four SMRT cells on the PacBio RSII using P6-C4 chemistry, at the UC Davis Genome Center (University of California, Davis).

### Quality control and transcriptome assembly

Short read technologies (Illumina MiSeq and HiSeq) and the PacBio Iso-Seq reads, which result from size-selected libraries ranging from 1000 to over 6000 nt, were included in both single and combined *de novo* assemblies. Seven MiSeq, 9 HiSeq, and 9 PacBio libraries were included. A total of 35 SMRT cells (1–4 SMRT cells per library) were sequenced and analyzed. The HiSeq and MiSeq Illumina reads were quality filtered and trimmed via Sickle (Joshi and Fass 2011) (v1.33, min. quality 35, min. sequence length 45 nt) and visually analyzed with FastQC (http://www.bioinformatics.babraham.ac.uk/projects/fastqc/). Quality trimmed Illumina reads from each library were independently *de novo* assembled with Trinity (Haas *et al.* 2013) (v.trinityrnaseq-r20140413p1, min. contig length 300 nt PacBio data were quality filtered (min. length 300 nt, read quality ≥ 0.7) and analyzed with the SMRT pipeline (https://github.com/PacificBiosciences/SMRT-Analysis). Raw reads were processed to obtain the circular consensus reads (CCS) and, additionally, CCS were subjected to an isoform level clustering step with ICE/Quiver, also provided through the SMRT pipeline (default parameters). Chimeric reads were evaluated with the RS_IsoSeq classify tool as the difference between total full-length and total full-length nonchimeric reads. PacBio results are provided for transcripts identified as full-length (Pa), and set of transcripts after ICE/Quiver for isoform level clustering: consensus sequences (Pb1), low quality polished sequences (Pb2), and high quality polished sequences (Pb3). For analysis of the number of full-length transcripts, sequences were queried against a local database containing curated plant protein sequences by means of USEARCH-UBLAST (v7.0.1090, *E*-value threshold of 1e−9) (Edgar 2010). Three types of hits were recorded: total hits (^1^H), hits covering 70% of the transcript (H2), and hits covering 70% of the transcript and 70% of the aligned protein (H3). These last two categories were used to estimate the proportion of potential full-length transcripts in the data. Rarefaction curves were generated by randomly selecting 1000 transcripts and analyzing mapped reads (see *Transcript abundance estimation* section) with the R (v3.3.0) package, Vegan (v2.3-4), to ascertain whether the sequencing depth and coverage were sufficient (Oksanen *et al.* 2016). Ribosomal RNA contamination among the assembled transcripts (before CDS identification) was assessed via BLAST (v2.2.29+, *E*-value 1e−9) against the SILVA database (release 04.04.2016) (Quast *et al.* 2013).

### Transcriptome annotation

Following assembly, coding DNA sequences (CDS) were identified with Transdecoder (Haas *et al.* 2013) (v.trinityrnaseq-r20140413p1) for both Illumina and PacBio CCS reads. Conifer protein sequences (*P. taeda* and *P. abies*), retrieved from PineRefSeq (http://treegenesdb.org/ftp/Genome_Data/genome/pinerefseq/Pita/) (Wegrzyn *et al.* 2014) and Congenie (ftp://plantgenie.org/Data/ConGenIE/Picea_abies/) (Sundell *et al.* 2015) projects, respectively, were used to train the machine learning component, and Pfam (v28.0) domain identification was used for CDS retention. High quality polished sequences from the ICE/Quiver clustering were also used for CDS identification with ANGEL (https://github.com/PacificBiosciences/ANGEL) using the same conifer sequences for training to complement the Transdecoder analysis. All CDS from Illumina and PacBio data were clustered at 95% sequence identity with USEARCH-UCLUST (v8.1.1861) (Edgar 2010) to generate a nonredundant set of transcripts. For functional annotation, the longest complete CDS from each transcript was subject to USEARCH-UBLAST to identify local alignments (v7.0.1090, *E*-value threshold of 1e−9 and a weak *E*-value of 0.0001) (Edgar 2010). NCBI’s RefSeq Protein (Release 69) (accessed Dec 2015), and the *Arabidopsis* protein database (TAIR, v10) were queried. Selection and assignment of the best annotation based upon the alignments was performed with the Eukaryote Non-Model Transcriptome Annotation Pipeline (enTAP v1.0, https://github.com/SamGinzburg/WegrzynLab, *E*-value 1e−5). Transcripts associated with bacterial, fungal, and insect contaminants were filtered as part of the annotation process. Gene Ontology (GO) (Ashburner *et al.* 2000) terms were assigned for Molecular Function, Biological Process, and Cellular Component with Blast2GO (v3.2.7, default parameters) (Conesa *et al.* 2005). MicroRNA (miRNA) annotation was analyzed with MIRENA (Mathelier and Carbone 2010) via previously identified miRNAs available in MirBase (v21) (Kozomara and Griffiths-Jones 2014). Over 800,000 transcripts lacking a CDS were used as input. miRNA precursors were identified, allowing up to two mismatches and a minimum MFEI index of −0.85 as a cutoff (Zhang *et al.* 2006a). Selection of high quality sequences was performed by manual inspection of RNA precursor secondary structures generated by ViennaRNA (Lorenz *et al.* 2011a) on the set of conserved miRNAs across land plants (Zhang *et al.* 2006b; Cuperus *et al.* 2011). Precursors were considered high quality if they met previously described miRNA structural requirements (Meyers *et al.* 2008).

### Evaluation of completeness

Completeness of the gene space was analyzed by following the single-copy orthologous approach deployed in the BUSCO pipeline (Simão *et al.* 2015) with default parameters and the plant reference set (950 orthologs). Assembled transcripts were also mapped against the *P. lambertiana* reference genome (v1.0) with Gmap (v2015-06-23) (Stevens *et al.*, 2016; Wu and Watanabe 2005). The gmapl version of the software was used due to the large assembled genome size. Mapping rate was calculated as the number of transcripts aligning at 98% of coverage and 98% of sequence identity, as well as 90% coverage/98% identity.

### Gene family analysis

The Markov cluster (MCL) algorithm analysis (v.12-068) (Enright *et al.* 2002), as implemented in TRIBE-MCL (Dongen and Abreu-Goodger 2012), was used to cluster the 385,329 protein sequences from 13 species into orthologous groups. Species included: *Glycine max* (37,388), *Ricinus communis* (28,113), *Populus trichocarpa* (36,393), *A. thaliana* (27,160), *Theobroma cacao* (28,136), *Vitis vinifera* (25,663), *Oryza sativa* (41,186), *Zea mays* (37,805), *Physcomitrella patens* (36,393), *P. lambertiana* (33,113), *P. taeda* (21,346), *P. abies* (19,607), and *P. glauca* (13,026). Angiosperm sequences were retrieved from the PLAZA (v3.0) set (Proost *et al.* 2015), pine sequences from the PineRefSeq project (http://dendrome.ucdavis.edu/ftp/Genome_Data/genome/pinerefseq/), and spruce sequences from the Congenie project (ftp://plantgenie.org/Data/ConGenIE/) (Sundell *et al.* 2015). All protein sequences were clustered at 95% identity with USEARCH-UCLUST. Subsequently, pairwise NCBI BLAST v2.2.29+ (*E*-value 1e−05) was run against the clustered set (Altschul *et al.* 1990). The negative log10 of the resulting blastp *E*-values was used to define the orthologous groups, and a moderate inflation value of 4.0 was selected. Following family assignments, Pfam domains were identified from the PLAZA (v3.0) annotations of the individual sequences. InterProScan (v.5.13-52.0; Hunter *et al.* 2012) was applied to those sequences obtained outside of PLAZA. Pfam and GO assignments with *E*-values < 1e−05 were retained. Families with protein domains classified as retroelements were removed. After functional assessment and filtering, custom scripts and Venn diagrams (http://bioinformatics.psb.ugent.be/webtools/Venn/) were applied to visualize gene family membership among species.

### Transcript abundance estimation

Transcript abundance estimation between samples without replicates was calculated with Gfold (v.1.1.2) (Feng *et al.* 2012). Treated samples were compared against their respective control: NaCl-treated root samples (NACLR) *vs.* untreated root (DCR); stem of methyljasmonate-treated plants (JASS) *vs.* stem of untreated plants (DCS); and stem tissue after wounding (WS) *vs.* stem of untreated plants (DCS). Among tissue types, reproductive tissues were compared with the basket sample (blend of needle, stem, and root). Quality filtered Illumina reads were mapped against the set of 33,113 assembled *P. lambertiana* transcripts with Tophat2 (v2.1.0) (Kim *et al.* 2013) with default parameters. Alignment (SAM) files were used as input for Gfold. A minimum fold change of 2.0 (-sc 2.0) was required for genes to be identified as differentially expressed. The expression table provided by Gfold for the complete set of 33,113 transcripts was used as input for labdsv (v1.8-9) R (v3.3.0) package (Roberts 2016) to perform the Principal Component Analysis (PCA). Overrepresented GO terms in differentially expressed genes were analyzed with Blast2GO (v3.2.7, default parameters) (Conesa *et al.* 2005).

### Analysis of Dicer gene family

DCL sequences were identified through functional annotations assigned via enTAP. Gene models corresponding to DCL *P. lambertiana* proteins were retrieved from the genome annotation (v1.0) (http://dendrome.ucdavis.edu/ftp/Genome_Data/genome/pinerefseq/Pila/v1.0/gene_models/) and transcriptome. USEARCH similarity searches were performed against *Arabidopsis* DCLs from GenBank (NM_001197952.1, NM_001202869.1, NM_001161190.2, and NM_122039.4) as well as protein domains identified by InterProScan (helicase, Dicer, PAZ, RNAseIII, and ds-RNA binding). Protein sequence alignments were generated with MUSCLE (v3.8.31) (Edgar 2004). Phylogenetic trees were generated with Fastree (v2.1.8) (Price *et al.* 2010) and visualized with FigTree (v1.4.1) (http://tree.bio.ed.ac.uk/). The redundant set of transcripts (before sequence clustering) and not the unique set (33,113 transcripts) was used for DCL analysis, in order to identify sequence variants and to provide additional evidence that the same or similar transcripts were sequenced from different tissues and/or libraries.

### Data availability

Assemblies are available in the TreeGenes database (http://treegenesdb.org/ftp/Genome_Data/genome/pinerefseq/Pila/v1.0/transcriptome/) and in NCBI as a TSA submission (GEUZ00000000). Raw reads, including the current draft sugar pine genome assembly, are available in NCBI BioProject PRJNA174450 (samples SAMN05256544, SAMN05256552, SAMN05271999, SAMN05272013, SAMN05272041, SAMN05272042, SAMN05272043, SAMN05272242, SAMN05272243, SAMN05282317, SAMN05282318, SAMN05282319, SAMN05282324, SAMN05282872, and SAMN05282873; SRA accession numbers for sequencing data SRR3689473, SRR3696256, SRR3696257, SRR3710655, SRR3712438 to SRR3712442, SRR3723920 to SRR3723927, SRR3724538, SRR3825176 to SRR3825202, and SRR3882733 to SRR3882738). Supplemental Material, Table S1 contains the tissue sample description and sequencing statistics; Table S2 lists the number of raw transcripts with similarity to ribosomal RNA; Table S3 lists number and types of splice variants identified; Table S4 contains all the statistically significant GO terms identified in differentially expressed transcript sets; Table S5 and Table S6 contain the number of proteins that compose the conifer-specific protein families and protein annotations, respectively; Table S7 and Table S8 contain the number of proteins that compose each *P. lambertiana*-specific protein family and protein annotation, respectively; Table S9 lists miRNA precursors; Figure S1 shows rarefaction curves of sequenced libraries; Figure S2 shows transcript length distribution of assembled transcripts where no CDS was identified; Figure S3 shows transcript length distribution for samples covered by different sequencing technologies; Figure S4 shows the contribution of each technology to improve the transcript completeness (extension of Figure 5); Figure S5 shows the number of splice variants provided by each technology in samples covered by several sequencing technologies (extension of Figure 6); Figure S6 shows plant species with the most protein sequence similarity to *P. lambertiana* transcripts; Figure S7 shows a transcriptome characterization by tissue samples. Figure S8 shows a treatment-specific *P. lambertiana* transcript analysis; Figure S9 shows PCA of sugar pine samples used for gene expression estimation; Figure S10 number of differentially expressed genes shared among several samples; Figure S11 shows a phylogenetic analysis of DCL proteins from *P. lambertiana* and several plant species; Figure S12 shows a gene expression analysis inferred from sequencing data of *P. lambertiana* transcripts codifying for DCL proteins; Figure S13 shows secondary structure from three *P. lambertiana* miRNA precursors; File S1 provides an extended description of the analysis of 2 cm female cones and female cones at the time of pollination for transcriptome diversity analysis; File S2 provides an extended description of the gene expression analysis.

## Results and Discussion

### Transcriptome sequencing and assembly

**Figure 1:**
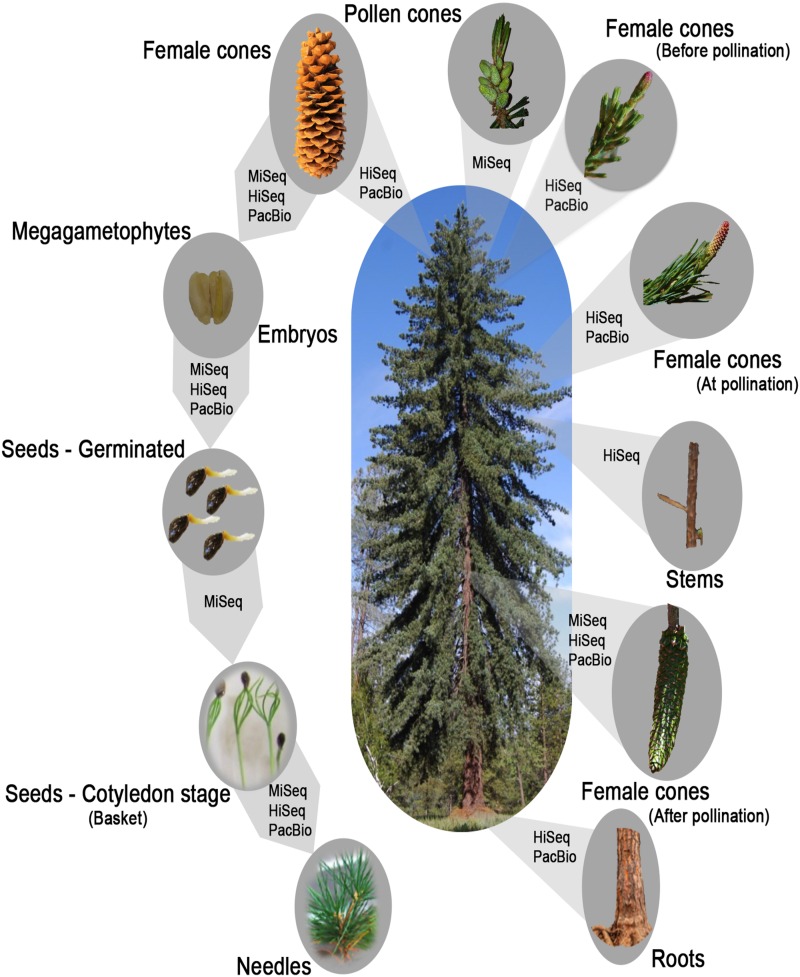
The selection of *P. lambertiana* tissues for transcriptome sequencing and the sequencing technologies applied.

A total of seven MiSeq, nine HiSeq libraries, and 35 PacBio SMRT cells corresponding to nine libraries from four samples were sequenced and analyzed, providing a set of 2.5 billion and 1.6 million Illumina and PacBio reads, respectively (Figure 1 and Table S1). A variety of tissues were included: vegetative (stem, root, and needle), reproductive (male and female cones and embryos), and various biotic and abiotic treatments. Select samples were represented by several technologies [embryo by all three platforms, 2 cm female cones, and female cones at the time of pollination by both HiSeq and PacBio]. Deep sequencing was obtained for each Illumina library with read totals ranging from 116 to 134 million for HiSeq, and 14 to 19 million for MiSeq. Over 100,000 transcripts were obtained from each *de novo* library assembly with an average length of 906 nt. Total unique genes ranged from 50K to 100K for HiSeq and 49K to 116K for MiSeq. The size-selected PacBio libraries represented 11 unique libraries (four of 1 kb, four of 2 kb, and three of 3–6 kb) in order to capture the full range of transcript sizes, and generated 60,000 raw sequences per SMRT cell. This yielded between 14K and 125K full-length and nonchimeric transcripts per library. The percentage of chimeric reads discarded ranged from 0.15 to 0.43% (Table S1). Transcripts had an average length of 1736, 1917, and 3570 nt for the 1, 2, and 3–6 kb size-selected libraries, respectively, revealing the effectiveness of size selection. Overall, quality was inversely proportional to read length, likely due to the fewer total passes to build the consensus sequence for the longest reads. SMRT tools provided an additional isoform level clustering step with ICE/Quiver producing three transcript sets: consensus isoforms (Pb1), low quality polished sequences (Pb2), and high quality polished sequences (Pb3). Clustering did not improve the length of the identified transcripts, but the high quality polished transcripts resulted in a set of sequences that performed well in terms of functional annotation and genome alignment.

Following the detection of CDSs from 1,087,300 assembled transcripts, 278,812 were clustered at 95% identity to provide a set of 33,113 unique, high quality, full-length transcripts, which ranged from 300 to 13,000 nt in length (Table 1). In the case of PacBio transcripts, more than one open reading frame was identified in the opposite direction in 6.79% of the sequences. After transcript selection (CDS identification and clustering), the percentage was reduced to 0.05%. Sequencing saturation of the libraries was estimated for each technology (Figure S1). HiSeq revealed the greatest saturation, MiSeq to a lesser extent, and PacBio reads did not reach complete saturation. Assembled transcripts with similarity to ribosomal RNA represented less than 0.1% for Illumina libraries and, at most, 3.8% for PacBio libraries (Table S2). Sequences related to transposons and other retroelements accounted for 24% of sequences, which were discarded. This is not in agreement with estimations of transposon content in conifer genomes, since not all elements are transcribed.

**Table 1:** Transcriptome statistics

Assembled Transcripts	Number of Sequences
Total transcripts	278,812
HiSeq	75,175
MiSeq	45,524
PacBio	158,113
Set of nonredundant transcripts	
Number of unique transcripts	33,113
Average length	1144
Shortest transcript	300
Longest transcript	13,236
N50	1386
Functional annotation statistics for the nonredundant set	
Annotated	30,839
Informative	26,568
Uninformative	3923
Unannotated	1243
Contaminants	1399

### Comparison of sequencing technologies

DNA sequencing represents one of the most significant technological revolutions in the past decade (van Dijk *et al.* 2014). Second generation technologies, as implemented by Illumina, have provided an increase in throughput at the cost of read length (25–300 nt) and quality compared to Sanger sequencing. Third generation technology, currently provided primarily through PacBio, provides lower throughput and lower quality reads at a higher price point but with significantly longer lengths (up to 20 kb) (Glenn 2011; Liu *et al.* 2012; Quail *et al.* 2012). In this study, germinated *P. lambertiana* seed (embryo) libraries (PacBio, MiSeq, and HiSeq) were evaluated for their overall contribution to accurate and comprehensive *de novo* assembled transcripts. Reads from each library were assembled independently and subsequently combined. The assemblies were analyzed across several metrics to determine the ability of deep Iso-Seq to replace second generation strategies. The analysis focused on both the individual transcripts (length, completeness, and mapping rates), as well as the whole transcriptome (coverage and diversity) to provide the first in-depth sequencing technology comparison in conifers.

#### Comparison of Illumina and PacBio transcriptome assemblies:

**Figure 2:**
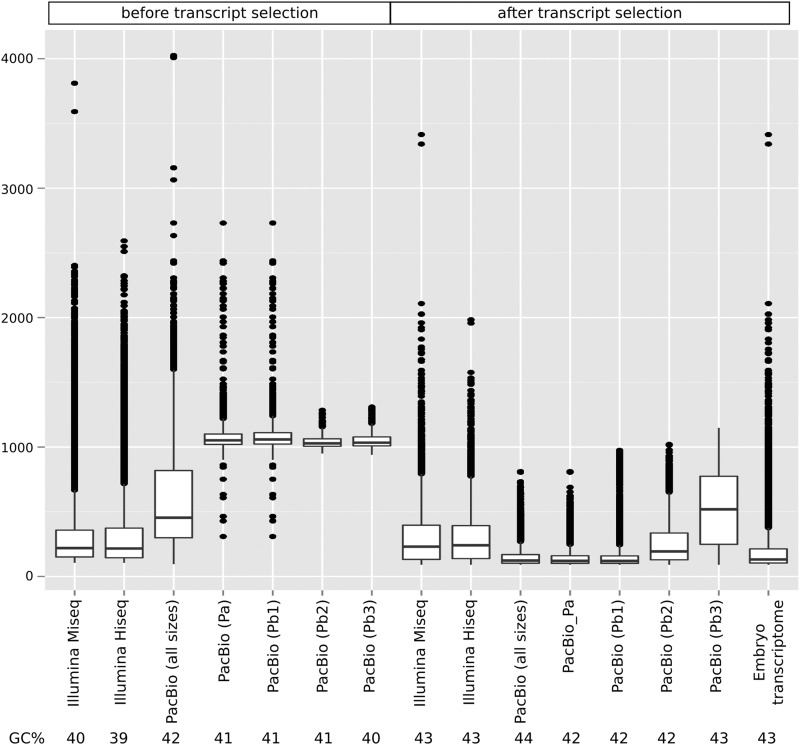
Transcript length distribution resulting from different assemblies of the embryo samples across the three technologies (HiSeq, MiSeq, and PacBio). Length of transcripts was used to build a box-plot distribution before and after transcript selection (CDS identification + clustering). PacBio results are provided for transcripts identified as full-length (Pa), and set of transcripts after ICE/Quiver for isoform level clustering: consensus sequences (Pb1), low quality polished sequences (Pb2), and high quality polished sequences (Pb3). Embryo transcriptome: combination of independent assemblies of Illumina and PacBio data and transcript selection (CDS identification + clustering). Average GC content of transcripts is shown in the bottom of the figure.

**Figure 3:**
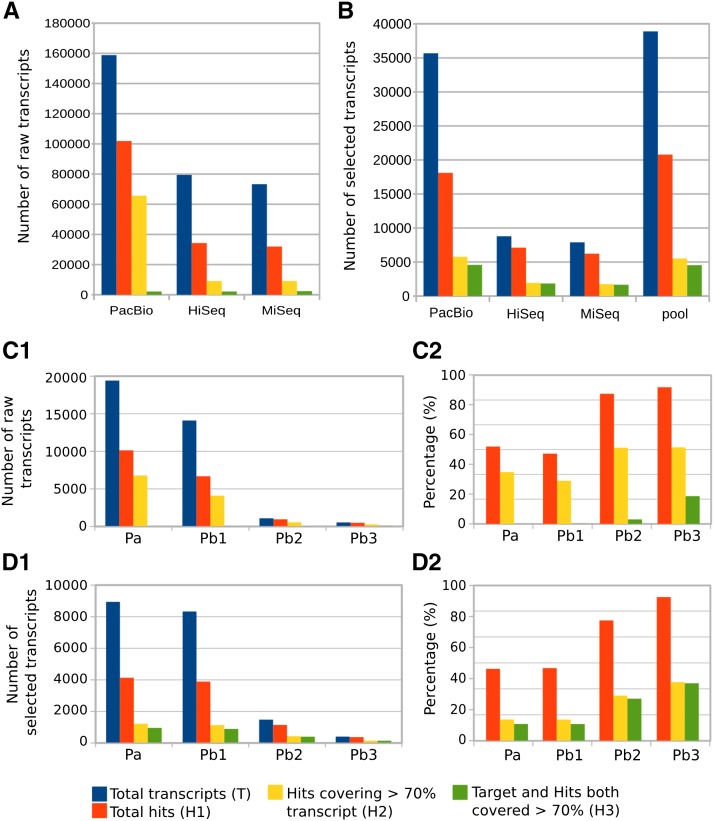
Transcript completeness analysis of different assemblies for embryo samples across the three technologies (HiSeq, MiSeq, and PacBio). Total number of sequences (T) were queried against NCBI RefSeq plant proteins by means of USEARCH-UBLAST. Three types of hits were counted: total number of hits (^1^H), hits covering 70% of the transcript (H2), and hits covering 70% of the transcript and 70% of the aligned protein (H3). (A) Raw assembled transcripts. (B) Sequences after transcript selection (CDS identification + clustering). (C1) Raw transcripts obtained with SMRT analysis for library Embryo_3–6 kb: transcripts identified as full-length (Pa), and set of transcripts after ICE/Quiver for isoform level clustering: consensus sequences (Pb1), low quality polished sequences (Pb2), and high quality polished sequences (Pb3). (C2) The same as C1, but expressed as percentage of sequences relative to the total number of transcripts (T). (D1) Sequences from (C1) after transcript selection (CDS identification + clustering). (D2) The same as (D1), but expressed as percentage of sequences relative to the total number of transcripts (T).

**Figure 4:**
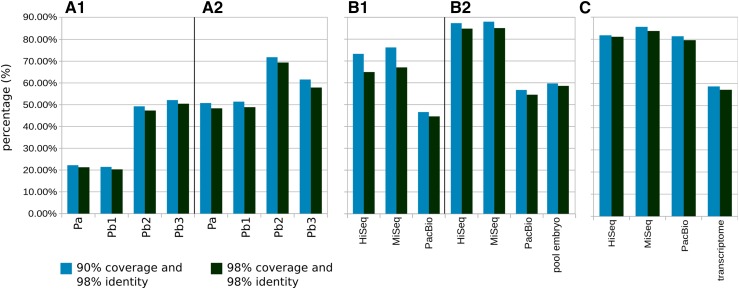
Mapping rates of different transcript sets to the *P. lambertiana* genome (v1.0). Sequences were aligned to the *P. lambertiana* genome and the percentage of mapped transcripts was calculated at two combinations of coverage and sequence identity. (A) Transcripts obtained from the SMRT analysis for embryo (3-6 kb size-selected): transcripts identified as full-length (Pa), and set of transcripts after ICE/Quiver for isoform level clustering: consensus sequences (Pb1), low quality polished sequences (Pb2), and high quality polished sequences (Pb3); before (A1) and after (A2) transcript selection (CDS identification + clustering). (B) Pool embryo: all size selected (PacBio), HiSeq, and MiSeq (Illumina); before (B1) and after (B2) transcript selection. (C) Complete transcriptome set.

Transcript length comparison of independently assembled reads demonstrated that PacBio overwhelmingly produces longer transcripts (Figure 2). In comparing the selected CDSs (trimmed CDS sets as defined by Transdecoder), PacBio yielded a larger number of complete CDSs than Illumina [8940 *vs.* 7892 (MiSeq) and 8782 (HiSeq) transcripts], in spite of starting with fewer reads. However, the length of PacBio transcripts was significantly reduced after high quality, full-length protein sequences were selected. The resulting processed lengths were similar to the processed Illumina transcripts (Figure 2). It is difficult to assess, given the bias introduced by angiosperm-dominated databases, whether conifers have longer CDS sequences than PacBio is able to detect or this technology is producing unlikely transcripts with no biological meaning. GC content of the different sequence sets did not show strong differences, but a slight increase was noted after transcript selection (from 39 to 42%) relative to raw transcripts, and for PacBio transcripts relative to Illumina (Figure 2). In some studies, PacBio has shown bias toward GC-rich sequences in genome sequencing (Quail *et al.* 2012). Among transcripts without a CDS, 43.5% of Illumina and 55.6% of PacBio transcripts were not aligned, respectively. It is interesting that, in the case of Illumina, the transcript length difference between mapped and nonmapped transcripts was small, but was larger in the case of PacBio (Figure S2). Less than 4% of transcripts were identified as full-length (70% of reciprocal coverage query:target) with either technology (Figure 3A). The same analysis after CDS selection yielded a significant improvement (as much as 21%) in the number of full-length transcripts (Figure 3B), revealing transcript selection as efficient for selecting potential full-length protein-coding transcripts. To evaluate the transcripts against the draft genome, the percentage of transcripts aligning at various coverage and identity combinations was calculated. Less than 50% of PacBio transcripts mapped to the genome compared to the assembled Illumina transcripts (> 70%) (Figure 4). Following transcript selection, close to 60% of PacBio transcripts aligned while nearly 90% of Illumina transcripts aligned. Some transcripts aligned to the end of two different scaffolds due to the fragmentation of the early draft genome assembly, contributing to a reduction of the mapping rates.

The single PacBio Iso-Seq embryo library (3–6 kb size selected) was selected to analyze each of the four outputs from the SMRT pipeline. After full-length transcripts (Pa in Figure 2) were assembled from raw reads, the software provides an optional isoform level clustering step to reduce isoform redundancy (Pb1 in Figure 2), and an additional sequence polishing step to improve quality (Pb2 and Pb3). The isoform level clustering step did not improve the length of the identified transcripts (Figure 2, lanes 4, 5, 6, and 7). Similar to the pool of all PacBio libraries (Figure 2, lanes 3 and 10), transcript selection of high quality proteins resulted in a reduction of transcript lengths (Figure 2, lanes 11, 12, 13, and 14). However, after transcript selection, longer lengths were achieved in the “polished” sets (Figure 2, lanes 13 and 14). When evaluated against characterized proteins, CDS selection increased the number of full-length sequences for each category (Figure 3, C and D). The total number of sequences decreased as the quality increased (Figure 3, C and D). When aligned to the *P. lambertiana* draft genome reference, the four sets followed the same trend. The best results were obtained after transcript selection and for the polished sequences (Figure 4A). In summary, ICE/Quiver polishing after isoform level clustering resulted in a drastic reduction in the number of final clustered and filtered sequences [*e.g.*, only 406 (2%) sequences were retrieved], but with significantly better performance in terms of quality (length, transcript completeness, and mapping rates).

#### Transcriptome coverage and diversity:

The 17,505 unique embryo transcripts generated from the combined HiSeq, MiSeq, and PacBio *de novo* assembled transcriptome were mapped against the *P. lambertiana* genome at 100% coverage and 90% identity. In total, 3846 transcripts did not map, 4410 mapped in more than two locations, and 9249 were single mapping units (SMUs) (one location in the genome). These SMUs were exclusively considered for downstream analysis. The complete set of transcripts for the embryo libraries (76,302 before clustering) were aligned to the genome with the same parameters, and those that overlapped with SMUs were selected. Of the 9249 SMUs, 4504 (49%), 3877 (42%), and 6883 (74%) were covered by HiSeq, MiSeq, or PacBio transcripts, respectively. These results revealed improved coverage by PacBio.

**Figure 5:**
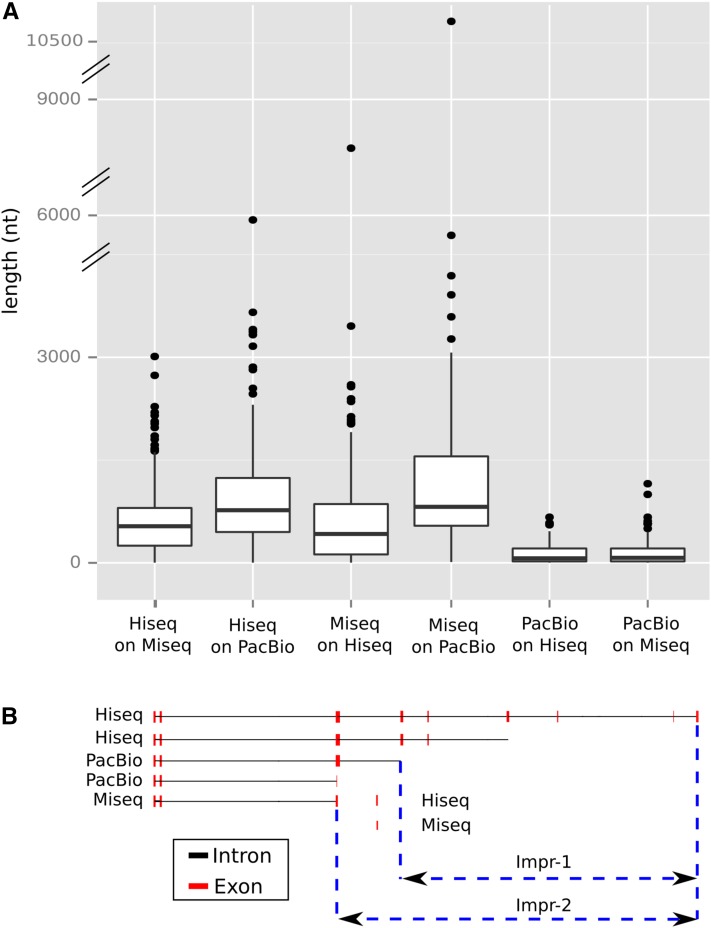
(A) Contribution of each technology to improve the coverage of the single mapping units (SMU) when it performed best. (B) Example of splicing variants identified and mapped on the same SMU (*P. lambertiana* transcript annotated as “embryo defective 2410 isoform protein” via enTap) for each technology. In this example, HiSeq performed as the best technology providing the longest splicing variant. Longest splicing variant of the other two technologies was selected to calculate coverage improvement (as the sum of exon sequence lengths, blue dashed lines, Impr-1 = PacBio, Impr-2 = MiSeq) of HiSeq technology over them [lanes 1 and 2 from (A)].

**Figure 6:**
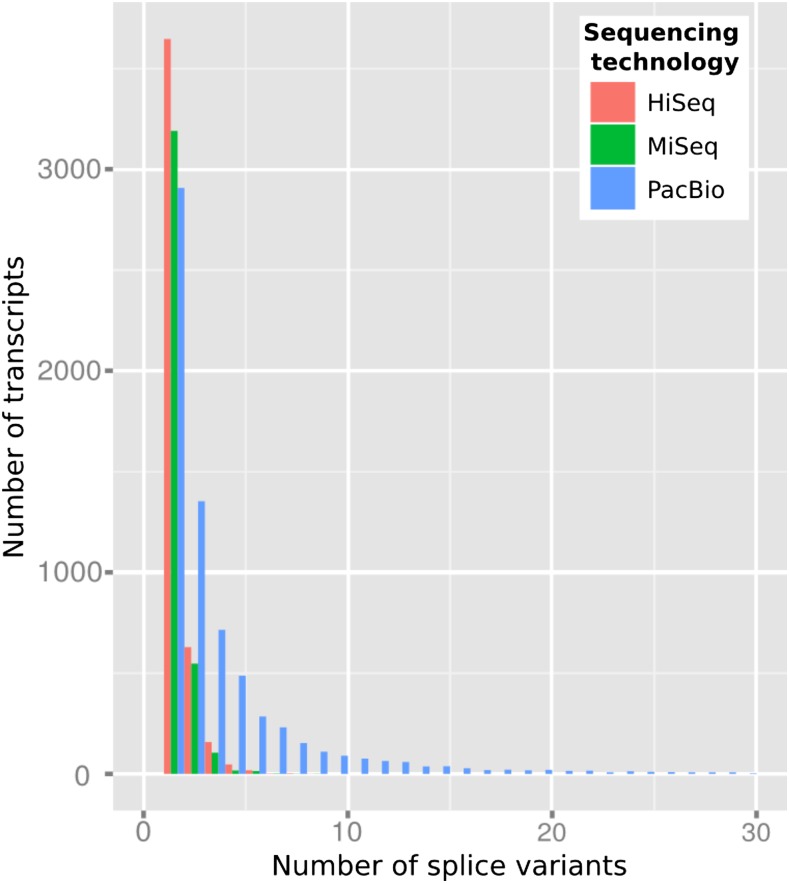
Number of splice variants generated across the three technologies (HiSeq, MiSeq and PacBio) in embryo.

A total of 1615 SMUs covered by all three technologies were evaluated on four different metrics. Examination of the longest splice variants revealed 1325 SMUs by HiSeq, 1191 by MiSeq, and 491 by PacBio (best result provided by one, two, or three technologies). Second, the number of SMUs where one single sequencing technology produced the longest splice variant was 251, 146, and 128 for HiSeq, MiSeq, and PacBio, respectively. Examination of transcript length distribution indicated that SMUs where PacBio was the best technology were shorter than their Illumina counterparts (Figure S3). Third, analysis of the contribution of each technology to the coverage of the SMU (where it was the longest transcript) was performed. For example, a single SMU with a HiSeq transcript of 1000 nt, a MiSeq of 600 nt, and a PacBio of 250 nt demonstrates that the HiSeq transcript improves the coverage by 400 nt relative to the MiSeq transcript, and 750 nt relative to the PacBio transcript. It is worth noting that the most significant improvements were observed for HiSeq and MiSeq transcripts relative to PacBio (Figure 5, lanes 2 and 4). Finally, the number of nonredundant splice variants was evaluated for each technology, for each SMU. Distribution across SMUs was improved in those transcripts originating from PacBio assemblies (Figure 6). For example, a set of 155 SMUs was covered by more than 30 variants as assembled with PacBio reads. On average (after the removal of outliers), the total number of splice variants per SMU was 1.6, 1.5, and 3.7 for HiSeq, MiSeq, and PacBio, respectively. A total of 92,300 splice variants were identified and characterized by type based on alignments to the reference genome. Overwhelmingly, length variants (alternative start or premature stop) were the most abundant, and intron retention was more abundant than exon skipping (Table S3).

Libraries from female cone tissue, sequenced with both PacBio and HiSeq, were used to evaluate transcriptome diversity, similar to the embryo libraries, to assess if the 3–6 kb size selected libraries can improve transcript length results for PacBio (Figure S3, Figure S4, Figure S5, and File S1). This analysis consisted of evaluating all size selected libraries and just the longest 3–6 kb library. Similar conclusions were reached in this analysis. PacBio libraries performed better in terms of coverage and splice variant detection, while Illumina libraries were advantageous for transcript length, longest splice variant, and contribution to improve the length of the SMU.

**Figure 7:**
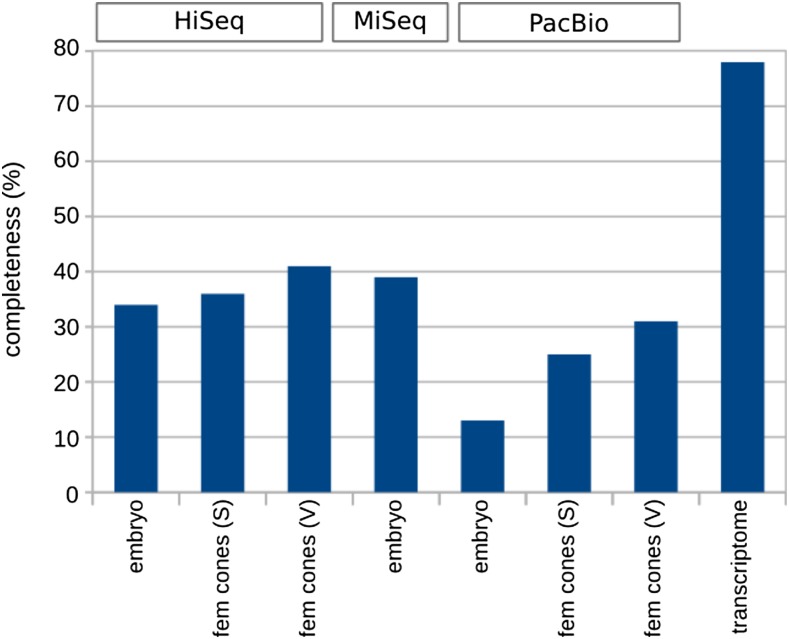
Transcriptome completeness analysis by BUSCO. Transcript sequences were compared to the plant set of single-copy conserved orthologs via BUSCO to estimate the percentage of completeness. Results are shown for sample embryo, 2 cm female cones, and female cones at time of pollination (lanes 1–7), corresponding all of them to the samples used in the sequencing technology comparison. The final column reflects the complete *P. lambertiana* transcriptome.

Transcriptome completeness was also analyzed with BUSCO for all three tissues used for sequencing technology review and evaluated in terms of technology. Lower completeness and higher variation (from 10 to 30%) between samples was achieved for PacBio libraries and better performance (up to 40%) for Illumina transcripts (Figure 7).

#### Overall comparison:

The low cost per base and error rate of the Illumina platforms drives the continued market preference. Despite the lower throughput and high error rate, PacBio Iso-Seq libraries were highly productive in terms of number of high quality transcripts. For example, 7892, 8782, and 8940 complete high quality CDS were identified in embryo samples from 29 million MiSeq reads, 230 million HiSeq, and 362K PacBio reads. PacBio yielded shorter assembled transcripts and sequence length was much improved on the Illumina platforms. This is in contradiction to the work of Xu *et al.* (2015) in *Salvia miltiorrhiza*, which reported longer PacBio transcripts compared to Illumina, although this comparison was performed prior to CDS selection. On the contrary, Dong *et al.* (2015) carried out a comparison in *Triticum aestivum* to determine length improvement of high quality (based on mapping rates) PacBio transcripts over previously annotated wheat gene models, and found minimal (45 nt on average) improvement. In our study, slightly better performance in sequence length was observed for HiSeq relative to MiSeq, and almost no difference in other metrics. Coverage of MiSeq libraries was lower than for HiSeq (only 7%), likely due to unusual HiSeq depth employed in this study (one lane per sample). MiSeq performed better than HiSeq in transcriptome completeness in the embryo sample as evaluated by BUSCO. This may suggest that the longer read length (300 nt PE) produced more representative sequences. PacBio produced the greatest number of splice variants, which is valuable given their role in regulating many biological processes in plant systems as well as the inability to accurately assess these in nonmodel systems. Recent studies in animal systems have benefited from long read technology for isoform detection (Thomas *et al.* 2014; Treutlein *et al.* 2014; Au *et al.* 2013; Sharon *et al.* 2013), while in plants (Dong *et al.* 2015; Xu *et al.* 2015), more efficient splice junction detection has been shown in technology comparisons (Li *et al.* 2014b). The promise of moving away from *de novo* transcriptome assembly of short reads and relying on third generation technologies has been proposed (Martin and Wang 2011). In our analysis, transcript yield of PacBio reached similar levels to Illumina, but transcript completeness was improved for the latter, suggesting the technology is not mature enough to replace the benefits of deep sequencing with short reads. A similar conclusion can be reached when comparing prices of the three technologies. MiSeq was 3 times more expensive than HiSeq (the least expensive), and PacBio 66 times. However, if we consider price per final transcript obtained instead of price per read, the difference is reduced and prices become much more similar. Accounting for all aspects, including price, technological, and biological concerns, a combination of both technologies is ideal for comprehensive and accurate transcriptome profiling.

### Transcriptome characterization

Among the 33,113 unique high quality full-length transcripts, 30,809 were functionally annotated with a protein from publicly available sequence databases. A total of 26,568 had a descriptive functional annotation (informative), 3923 were uninformative (annotated as hypothetical, predicted, or otherwise noncharacterized proteins), and 1399 were strongly associated with fungal, insect, or bacterial sequences and removed from subsequent analysis. A total of 1243 remained unannotated, representing artifacts or potential novel conifer-specific proteins. In spite of not being annotated, at least one protein domain was identified in all (as required during selection of the CDS). Of these 1243, 351 contained a DUF-like domain (domain of unknown function), the most abundant occurrence labeled as DUF4283 (Table 2). A total of 189 transcripts contained a domain similar to cellulose synthases (PF03552). Proteins associated with cellulose metabolism were also identified in the gene family analysis as specific to *P. lambertiana*. Additionally, 94 X-box-related transcription factors were identified, which is expected due to the high specificity of these proteins for binding DNA (likely specific to *P. lambertiana*). When aligning the complete set of transcripts to characterized proteins, *A. thaliana* and *V. vinifera* dominated the annotations (Figure S6). The transcriptome was evaluated for completeness with BUSCO and over 78% of the 950 unique orthologs conserved across land plants were identified.

**Table 2:** Most abundant protein domains identified in nonannotated *P. lambertiana* transcripts

No. of Transcripts	Protein Domain	Domain Description
189	PF03552	Cellulose_synt
150	PF00098	zf-CCHC
81	PF14111	DUF4283
56	PF01535	PPR
48	PF00931	NB-ARC
43	PF00240	Ubiquitin
39	PF00560	LRR_1
27	PF00400	WD40
26	PF13504	LRR_7
26	PF00069	Pkinase

PF, Pfam database.

Expression profiles from several distinct tissue libraries were compared and unique transcripts were estimated. It is worth noting that the majority of unique sequences were expressed in female reproductive tissue (samples S, V, and M together, Figure S7A). Also, few unique transcripts were identified in basket stage tissues (Figure S7A) when compared to the other vegetative tissues, as expected, since this is a pool of cotyledons, stems, and roots. When the three vegetative tissues were compared to reproductive tissues, basket, and embryo, larger differences were observed for reproductive tissue (Figure S7B). Since this deep sequencing represents a single individual, transcripts that clustered with sequences from the same library were considered to be library-specific gene products (Figure S8A). This produced a range from 199 transcripts (basket) to 3482 transcripts (female cone at the time of pollination, sample V, Table S1). Interestingly, the female cones (2 wk before pollination) (sample M, Table S1), had a similar number of unique sequences to other vegetative tissues, when compared with other female cones samples (V and S, Table S1). The latter two were in a more developed stage of differentiated cone tissue. In total, 14,718 transcripts were shared by different libraries.

The lack of replicates in this study hampers confident identification of differentially expressed genes. However, preliminary evaluation of this can contribute to tissue characterization and provide insights into the biological processes underlying the individuals sampled. Treated samples have been compared to their respective untreated control (see *Materials and Methods*), and reproductive tissue has been compared to the basket stage seedling sample, as a mix of vegetative (needle, root, and stem) tissue. Number of reads mapped on each transcript was used as an estimate of RNA accumulation. Expression profiles of all transcripts (in each library) were used for a PCA (Figure S9), where samples corresponding to reproductive tissue grouped on the left half of the plot, and vegetative tissue samples on the right, with the notable exception of Basket samples. This is likely a result of the combined tissues at the early “basket” stage of development. PCA results confirmed that Basket samples were the least informative considering both transcript uniqueness and transcript accumulation. Female cones at time of pollination (V samples) represented the greatest transcript richness (uniqueness, see above) and also distinctive RNA accumulation profiles. Stem (red circle) and root (green circle) samples clustered together, showing smaller transcriptomic changes after treatments (NaCl, wounding, or jasmonate) than those occurred consequence of developmental processes. Interestingly, there is a parallel PCA component of the same sense from healthy to treated tissue for both stem and root samples. On average, 5958 transcripts were identified as differentially expressed in each sample with a fold change > 2.0, and shared genes among the samples compared. Following the same trend, jasmonate-treated and injured tissue shared more differentially expressed transcripts than NaCl-treated samples (Figure S10). The role of jasmonate in both pathogen defense and wounding response might explain this observation. Embryonic tissue shared only a few differentially expressed genes with the three vegetative tissues, and more similarities were found between embryo and reproductive tissues as expected (Figure S10). Enriched GO terms identified in the differential expression comparisons, included: defense response (jasmonate-treated samples), response to stress and cell wall modification (tissue after wounding), ATPase and osmosensor activities (NaCl-treated samples), and regulation of developmental processes (reproductive tissue) (Table 3 and Table S4). Despite experimental limitations, the identified differentially expressed genes were consistent with the underlying biology of the tissues and treatments (detailed analysis in File S2).

**Table 3:** Summary of GO terms overrepresented in differentially expressed *P. lambertiana* genes

GO-ID	Term	Category	FDR
NACLR			
GO:0042555	MCM complex	C	0.049359
GO:0043168	Anion binding	F	7.84E−18
GO:0005524	ATP binding	F	2.14E−09
GO:0016887	ATPase activity	F	0.004976
GO:0005034	Osmosensor activity	F	0.046233
GO:0010817	Regulation of hormone levels	P	6.04E−06
GO:0048767	Root hair elongation	P	0.006801
GO:0009809	Lignin biosynthetic process	P	0.023398
JASS			
GO:0010583	Response to cyclopentenone	P	0.007481
GO:0043207	Response to external biotic stimulus	P	0.021576
GO:0051707	Response to other organism	P	0.021576
GO:0051567	Histone H3-K9 methylation	P	7.68E−09
GO:0042742	Defense response to bacterium	P	0.028652
GO:0010476	Gibberellin-mediated signaling pathway	P	0.011213
GO:0042221	Response to chemical	P	1.90E−07
Wound			
GO:0006950	Response to stress	P	8.39E−09
GO:0006952	Defense response	P	2.54E−06
GO:0005911	Cell–cell junction	C	0.000132
GO:0030855	Epithelial cell differentiation	P	0.010485
GO:0060429	Epithelium development	P	0.017064
GO:0042545	Cell wall modification	P	0.035399
Reproductive tissue			
GO:0009751	Response to salicylic acid	P	0.002236
GO:0010333	Terpene synthase activity	F	6.87E−14
GO:0048506	Regulation of timing of meristematic phase transition	P	0.000373
GO:0007389	Pattern specification process	P	0.002009
GO:0009955	Adaxial/abaxial pattern specification	P	0.008520
GO:0007165	Signal transduction	P	1.66E−17
GO:0050793	Regulation of developmental process	P	2.67E−05
GO:0010476	Gibberellin-mediated signaling pathway	P	0.000174

GO-ID, Gene Ontology identifier; FDR, false discovery rate; NACLR, NaCl-treated; MCM, minichromosome maintenance protein complex; C, cellular component; F, molecular function; ATP, adenosine triphosphate; P, biological process; JASS, methyljasmonate-treated.

### Gene family analysis

**Figure 8:**
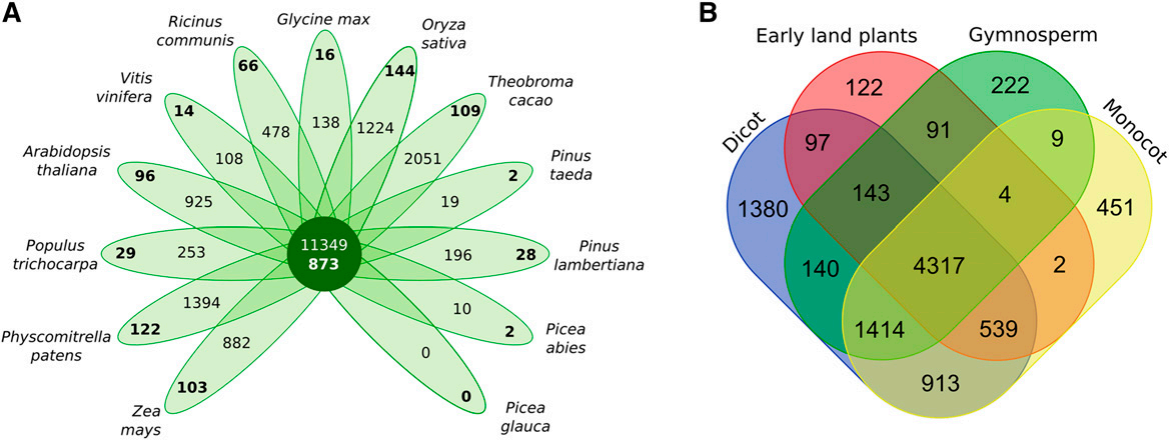
Results of the TRIBE-MCL analysis to identify orthologous proteins and gene families. (A) Number of species-specific proteins and families (bold). (B) number of protein families shared by different species grouped in main classes.

A total of 51,475 families out of 13 species were retrieved from the gene family analysis implemented in TRIBE-MCL. Of these, 9844 contained at least five protein members after filtering for retroelements. A total of 731 were composed of proteins from a single species and 9113 from two or more species (Figure 8A). Among conifers, the largest number of species-specific families was observed in *P. lambertiana* and the fewest in *P. glauca*, likely influenced by the varying transcript resources available for each species. A large number of proteins were shared by all species (11,349). Conservation among protein families was also compared across species grouped in four categories (bryophyte, gymnosperm, monocot, and dicot, Figure 8B). The largest number of shared families were those present in all four groups (4317). Both early land plants and gymnosperms shared more families with dicots than with monocots. Only 222 families were found unique in conifers: 4 unique to the genus *Picea*, 36 unique to *Pinus*, and 28 unique to *P. lambertiana*. Conifer and *P. lambertiana*-specific families and protein annotations are provided in Table S5, Table S6, Table S7, and Table S8. The largest family (74 proteins with 12 from *P. lambertiana*) was composed of transferases and uncharacterized proteins, revealing potential novel proteins. An abundant family composed of *mTERF* transcription factors (3 families comprising 87 proteins, 25 from *P. lambertiana*) play important roles in plant growth, development, and abiotic stress tolerance, based on characterization in *Arabidopsis* (Kleine 2012). Little is known about the molecular mechanisms of *mTERF* that control transcription of the mitochondrial and chloroplastic genomes, but the high content and the presence in the conifer-specific set suggest specific roles in gymnosperms. WRKY transcription factors were also abundant (4 families, 68 proteins, 21 from *P. lambertiana*), known as key regulators of many processes, including responses to biotic and abiotic stresses, senescence, seed dormancy, seed germination, and plant responses to pathogens (Rushton *et al.* 2010). F-box proteins known to be subunits of the E3 ubiquitin ligase aggregations named as the SCF quaternary complex (SKP1, Cullin1, F-box protein, and Rbx1, Zheng *et al.* 2002) were also identified as one of the most abundant families specific to conifers (8 families in total, 105 proteins, 35 from *P. lambertiana)*. In the *P. lambertiana*-specific set, two families containing proteins related to cellulose metabolism attracted attention, due to the potential connection to basal biology of a woody species. Among families shared by other species but potentially expanded in conifers, were two comprised of ATP binding proteins with large number of isoforms (787 members in *P. lambertiana*, and 390, 522, and 98 in *P. abies*, *P. taeda*, and *P. glauca*, respectively).

#### Characterization of the Dicer protein family:

Conifers have a distinguishing feature with regard to gene silencing and small RNA (sRNA) biogenesis in their unique 24-nt sRNA profile, which are associated with epigenetic processes and control of repetitive element proliferation (Matzke and Mosher 2014). The peculiar sRNA profile and large genomes with TE content reaching 80% raises questions about the involvement of the sRNA machinery in conifer genome expansion. Key components of this pathway include specialized members of RNA-dependent DNA polymerase, RNA-dependent RNA polymerase, Argonaute, and DCL proteins (Huang *et al.* 2015; Matzke and Mosher 2014), the latter involved in the biogenesis of sRNAs. In addition to plant development and abiotic stress, a link between DCLs and plant pathogen response exists, at least for viruses and bacteria (Matzke and Mosher 2014). There are four different DCL proteins characterized in *Arabidopsis*. DCL3 is primarily responsible for the epigenetic pathway. This number varies in other plants such as poplar and rice (Margis *et al.* 2006). In spite of initial reports, it is generally accepted that the 24-nt-DCL3 pathway exists in conifers, but with spatial and/or temporal peculiarities. Transcriptomic studies in pine and larch have noted that 24-nt sRNAs are restricted mainly to reproductive tissues and are decreased or even absent in vegetative tissues (Nystedt *et al.* 2013; Zhang *et al.* 2013; Niu *et al.* 2015). 21-nt sRNAs are associated with repetitive content in the Norway spruce genome (Nystedt *et al.* 2013) and conifer-specific DCL1 variants have been described (Dolgosheina *et al.* 2008).

#### Canonical plant DCLs shared by conifers:

In the *P. lambertiana* transcriptome, 12 transcripts were identified with sequence similarity and domain topology matching DCL features. Among these, six were supported by gene models in the draft genome sequence (Stevens *et al.*, 2016). These sequences were combined with plant DCL proteins to perform a phylogenetic analysis (Figure S11), including four conifers (*P. taeda*, *P. abies*, *P. glauca*, and *P. tabuliformis*), a monocot (*O. sativa*), a dicot (*A. thaliana*), *Amborella trichopoda* because of its phylogenetic position near the base of the flowering plants lineage, and *P. patens* (Bryophyta) and *Selaginella moellendorffii* (Lycopodiophyta) as model organisms of ancient land plants. The last two species have an additional interest because 24-nt small RNAs have been sequenced in *P. patens*, demonstrating the basal origin of the pathway, but they are weakly expressed compared to 21-nt sRNAs (Banks *et al.* 2011; Coruh *et al.* 2014). The proportion of 23–24-nt sRNAs relative to the 21-nt class is also reduced in the sporophyte of *S. moellendorffii*, where their expression is mostly limited to the gametophyte (Banks *et al.* 2011). It is worth noting that *S. moellendorffii*, in spite of a similar genome size and organization to *Arabidopsis*, has an increased repeat content and abundant LTR retrotransposons (Banks *et al.* 2011).

In the phylogenetic analysis, all conifers and the selected plant sequences grouped according to the four main classes of DCLs described to date (Figure S11). Two *P. lambertiana* sequences represented by two nonoverlapping gene models clustered with DCL3 proteins from other species, providing further evidence of its presence in gymnosperms. *P. patens* and *S. moellendorffii* have been reported to have no members for DCL2 (Axtell *et al.* 2007). Accordingly, we did not identify this DCL in these species. No DCL2 counterpart for *P. lambertiana* was identified, but it was found in sequences from *P. abies*. DCL2 orthologs from *P. tabuliformis* have also been reported, indicating that all four DCLs are present in most conifers. The absence of DCL2 in *P. lambertiana* might be due to misrepresentation in the transcriptome, although other studies have failed to find DCL2 in specific species of the gymnosperm order Gnetales (Ma *et al.* 2015). Investigating the needle transcriptomes of other white pines of which *P. lambertiana* is a member, *P. albicaulis* pine contained a high quality version of DCL2, while *P. flexilis* and *P. monticola* did not.

#### Conifer-specific set of DCL1 proteins:

**Figure 9:**
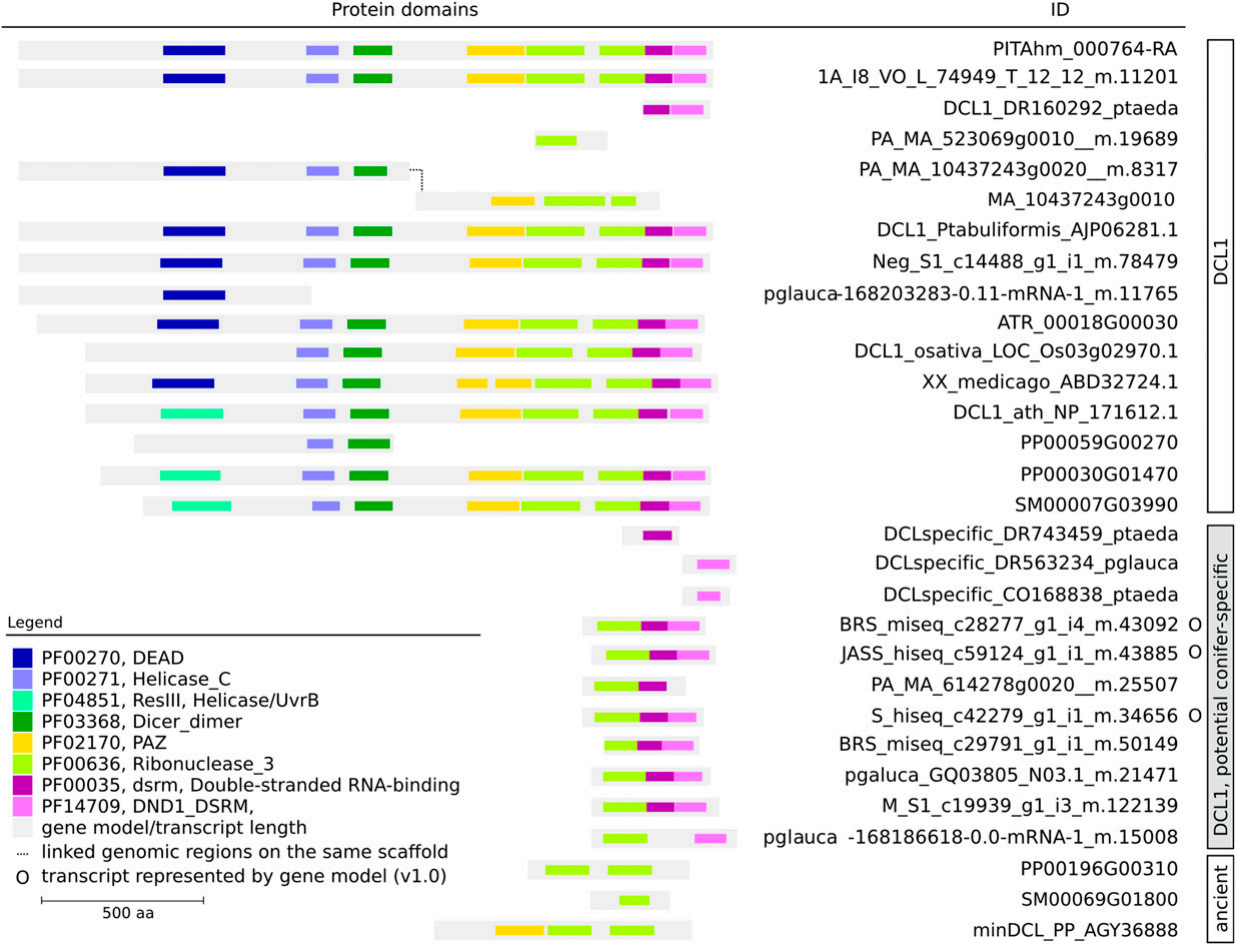
Protein domain topology of DCL1 proteins from *P. lambertiana* and several plant species, including three conifers [*P. taeda* (Ptaeda), *P. abies* (Pabies), *P. glauca* (Pglauca), and *P. tabuliformis* (Ptabuliformis)], a monocot [*O. sativa* (Osativa)], a dicot [*A. thaliana* (Athaliana)], *A. trichopoda* (*Atrichopoda*), *P. patens* (Ppatens), and *S. moellendorffii* (Smoellendorffii). DCL1, Dicer-like 1.

The DCL1 sequences split into two independent clusters, one grouping contained the canonical DCL1 protein from *Arabidopsis* and other plants, while the other encompassed some *P. lambertiana* transcripts and the conifer members identified as potentially specific by Dolgosheina *et al.* (2008). This also included some new sequences originating from the *P. glauca* and *P. abies* genome projects. All DCL1 sequences were further explored for protein domain architecture (Figure 9). Most of the non-conifer specific sequences had a canonical DCL1 architecture (two helicase, one Dicer, one PAZ, two RNAseIII, and two ds-RNA binding domains, from N- to C-terminus). *P. lambertiana* and *P. taeda* DCL1s were complete, as well as those from *P. tabuliformis*, the remaining angiosperms, *P. patens*, and *S. moellendorffii*. The *Picea* DCL1s were not complete. For *P. abies*, one locus (MA_523069g0010, Figure 9) was located in a small scaffold. The two additional sequences (MA_10437243g0010 and MA_10437243g0020, Figure 9) corresponded to complementary DCL1 parts located in two consecutive gene models on the same scaffold, which is likely a fragmented gene model. For *P. glauca*, no additional models within the range of the one identified were found. Previously identified conifer-specific DCL1s, as well as the remaining conifer sequences used in this study, were represented by a portion of a complete DCL1 sequence (1–3 domains). They lacked the N-terminus and the PAZ domain, but had conserved RNAseIII and dsRNA-binding domains (Figure 9). This result may be due to incorrect gene models, or might represent a unique function. The prevalence of pseudogenes and the fragmented genome assemblies in conifers complicates the determination of whether these conifer-specific sequences are artifacts derived from functional DCLs. For example, the gene models corresponding to the three short DCL1 transcripts with genome representation were surrounded by abundant TEs, which can be indicative of pseudogenes. However, the high quality transcripts represented by high quality gene models (*e.g.*, DCL3) were flanked in a similar manner. It is worth noting that PacBio data were not particularly informative for the identification of these sequence variants.

#### DCL1 protein variants in ancient plants:

Sequences from *S. moellendorffii* did not group with the DCL1 conifer-specific set, providing no evidence of shared genetic elements at this level with conifers. However, a protein sequence of similar features from *P. patens* was identified and clustered out of both DCL1 clades (conventional and conifer-specific, Figure S11 and Figure 9), suggesting a potential common origin for all species for these shortened DCL-like sequences. This sequence corresponds to a short variant of DCL1 recently characterized in *P. patens* and identified as MINIMAL DICER-LIKE (mDCL) (Coruh *et al.* 2015). This gene lacks the N-terminal helicase domain of DCL proteins, and has only PAZ and RNAseIII domains. The mDCL is specifically required for 23-nt siRNA accumulation associated with genomic repetitive elements. Mutant analysis showed a dependence of this protein on DCL3 for generating the complete set of siRNAs (Coruh *et al.* 2015). Phylogenetic resolution of this protein remained unclear, although it clustered with DCL1 sequences in spite of its association with siRNAs. In this study, it also clustered along with DCL1-like sequences from other species (Figure S11). We were able to identify a truncated version of *S. moellendorffii* DCL with only a RNAseIII domain and with sequence similarity to DCL1s that clustered alongside mDCL1, both basal to the overall DCL1 lineage. It has been suggested that shortened versions of DCLs might arise frequently during evolution (Coruh *et al.* 2015). For example, truncated versions of DCLs, which lack the N-terminal helicases and the PAZ domain (similarly to those identified in conifers), also have been described as functional in other non-plant organisms (Malone *et al.* 2005). The link between the shortened proteins and the conifer-specific set remains elusive, but these data suggest that an ancient mDCL from *P. patens* could have evolved through lycophytes and gymnosperms and not through angiosperms. Cloning and experimental characterization of the truncated conifer-specific DCL1 proteins is needed to determine if they are functional, but experimental data reported on mDCL in *P. patens* and other species supports the idea that complete domain topology of canonical DCL1 is not a requirement.

#### Expression analysis of DCL transcripts:

Expression analysis indicated tissue specificity for both canonical and conifer-specific DCLs. The transcript potentially coding for conventional DCL1 was ubiquitously expressed across all samples analyzed (Figure S12A). A similar profile was observed for one transcript coding for DCL4. The other two DCL4s were practically not expressed in any tissue, but were observed initially in cone samples. DCL3, which is involved in 24-nt sRNA biogenesis, was represented by three *P. lambertiana* transcripts, primarily expressed in reproductive tissues: one transcript slightly expressed in embryo, one in early female cones, and the third in pollen and highly overexpressed in embryo (Figure S12A). Conifer-specific DCL1 transcripts had a mix of profiles (Figure S12B). One was virtually not expressed, the other ubiquitously expressed, and the last had a differential profile among reproductive tissues. The most interesting profile was transcript BRS/miseq/c28277_g1_i4|m.43092, which was highly overexpressed in embryo with a similar profile to Basket/c18190_g1_i2|m.24310 (conventional DCL3-like protein). Experimental validation of the DCL3 protein and this truncated variant of DCL1 is necessary to confirm functional association with a similar mechanism reported for *P. patens*.

### miRNA precursor identification

**Figure 10:**
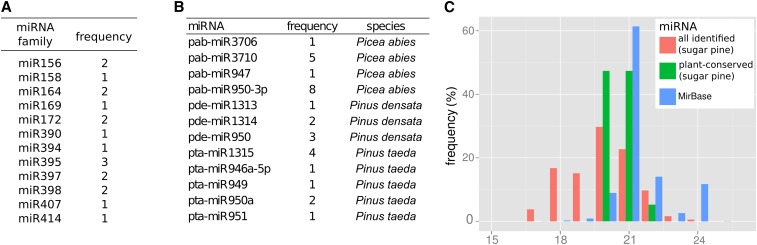
Computational prediction of mature microRNA (miRNA) sequences from precursors identified in *P. lambertiana* transcripts corresponding to plant-conserved (A) and nonconserved conifer-related (B) miRNA families. (C) Length distribution of identified mature miRNAs.

In total, 185 potential miRNA precursors were identified. None of these had an exact match to sequences deposited in MirBase as all contained one or two mismatches. In examining the size distribution of the mature predicted mRNAs, only one 24-nt sequence (0.5%) was identified (Figure 10C). A low frequency of 24-nt small RNAs (involved in transposon control in angiosperms) has been reported in gymnosperms (Nystedt *et al.* 2013; Zhang *et al.* 2013; Niu *et al.* 2015). The huge genome of *P. lambertiana* is primarily composed of TEs and the observation here suggests additional support for the hypothesis. The lack of targeted small RNA sequencing data in this study hampers validation of identified mature miRNA sequences. To accommodate this, we considered only those that contained a mature miRNA with sequence similarity to those most conserved among plants (49 precursors). Of these, 19 aligned to the core conserved plant miRNAs (Figure 10A) and 30 specifically to other conifers (Figure 10B). In addition, the RNA secondary structures have been manually reviewed to select precursors satisfying miRNA structural requirements (Figure S13A). Long precursors with strongly negative MFEI indexes (Figure S13B) were flagged as low quality as they resemble the structure of fold-back retrotransposons. Finally, multiple miRNA predictions on the same transcript corresponding to both strands of the miRNA duplex were collapsed, yielding a total of 37 and 9 high and low quality precursors, respectively (Table S9).

Precursor lengths ranged from 60 to 307 nt (125 nt in average), while source transcripts ranged from 246 to 2880 nt (1008 nt in average). Twenty-seven precursors successfully mapped to the *P. lambertiana* genome (Stevens *et al.*, unpublished results). Two sets of precursors (PILAmiRNA_026 and PILAmiRNA_007, Table S9) were located in the same scaffold, which were further explored for potential miRNA clusters codified in polycistronic transcripts. Precursors contained on the same transcript provide information about coexpressed miRNAs in the same family or even different families. PILAmiRNA_007 corresponded to miR1313-like precursors, which were located 120 kb apart, so not further considered, but PILAmiRNA_026 corresponded to 2 miR1314 precursors placed only at 326 nt apart, suggesting a cluster (Figure S13C). However, the source transcript aligned only to the first precursor, questioning whether the transcript is complete, the second precursor is expressed independently, or the second *locus* represents a nonfunctional region. The mature miRNA contained a nucleotide variant at position 13 relative to the sequence predicted by Mirena on the second transcript-supported precursor, suggesting a mutation.

The small number of precursors identified from the large transcriptome resource can be attributed to the short life span of primary miRNAs (Song *et al.* 2007). Sequencing data were used to estimate precursor accumulation, and differences in the level of expression were observed among miRNA families. The most abundant were miR156 and miR172 (seen in all samples and primarily in reproductive tissue). These miRNAs are conserved across nearly every plant species (Montes *et al.* 2014). One and two precursors were sequenced for miR156 and miR172, respectively. In contrast, the nonconserved miR950 showed moderate accumulation, mostly in stem samples, but 13 precursor variants were sequenced. The contrasting different ratios between level of expression and number of precursors detected in these three miRNA types serve as an example that different processing rates for different miRNA families might occur. miR950 has been characterized in *P abies*, *P taeda*, and *Pinus densata*, but is absent in the remaining plant species in miRBase, suggesting conifer-specificity. It has, however, been reported in flower buds and fruits in *Citrus sinensis* (Song *et al.* 2012). It has been suggested that its primary targets are NB-LRR genes, potentially as a source of phased secondary small interfering RNAs (Xia *et al.* 2015; Zhai *et al.* 2011). The lack of conservation across plants and the high number of precursor variants detected here may indicate an important role in conifers, and unique processing rates for this miRNA.

### Conclusions

This study characterizes the transcriptome of *P. lambertiana*, expanding the scarce genomic resources available for the subgenus Strobus. Due to inherent technical challenges in conifer genome assemblies, these resources are essential to provide insight on the complete gene space. Among the prevalent pseudogenes and TEs, annotation of true gene models is hindered without transcriptomic evidence. With this resource, we also provide the first computational identification of miRNAs in *P. lambertiana*, and, related to gene silencing, undertake an exploration and comparative analysis of DCL and DCL-like proteins. This is an outstanding question in gymnosperm biology, since several conifer-specific DCL variants are under investigation. Expression analysis derived from sequencing data further supports a biological role of these variants. The results presented here highlight the peculiarities of this pathway in conifers and identifies similarities with ancient land plants. From a technical perspective, we have used PacBio’s Iso-Seq long read strategy for the first time in a conifer to improve the accuracy of transcript construction. The detailed short and long read technology comparison provides perspective and recommendations for those generating transcriptomic resources in nonmodel species.

## Supplementary Material

Supplemental Material
