# Supplementary material for: Assessing the Gene Content of the Megagenome: Sugar Pine (Pinus lambertiana)
Source: G3 (Bethesda). 2016 Oct 31;6(12):3787–802. doi: 10.1534/g3.116.032805 (PMC5144951; doi:10.1534/g3.116.032805)
Supplement: Supplemental Material [file supp_6_12_3787__index.html]

Assessing the Gene Content of the Megagenome: Sugar Pine (Pinus lambertiana) — Supplemental Material 

# Assessing the Gene Content of the Megagenome: Sugar Pine (*Pinus lambertiana*)

## Supplemental Material for Gonzalez-Ibeas, *et al*, 2016

**Files in this Data Supplement:**

- File S1 - An extended description of the analysis of 2 cm female cones and female cones at the time of pollination for transcriptome diversity analysis. (.pdf, 53 KB)
- Figure S8 - Treatment-specific *P. lambertiana* transcripts analysis. (.pdf, 511 KB)
- Figure S9 - Principal component analysis (PCA) of sugar pine samples used for gene expression estimation. (.pdf, 114 KB)
- Figure S10 - Number of unique and shared differentially expressed transcripts for stressed tissues, embryo samples and pooled reproductive tissues (cones and pollen). (.pdf, 143 KB)
- Figure S11 - Phylogenetic analysis of DCL proteins from *P. lambertiana* and several plant species, including three conifers (*Pinus taeda* (Ptaeda), *Picea abies* (Pabies), *Picea glauca* (Pglauca) and *Pinus tabuliformis* (Ptabuliformis)), a monocot (*Oryza sativa* (Osativa)), a dicot (*Arabidopsis thaliana* (Athaliana), *Amborella trichopoda* (Atrichopoda), *Physcomitrella patens* (Ppatens) and *Selaginella moellendorffii* (Smoellendorffii). (.pdf, 2.95 MB)
- Figure S12 - Gene expression analysis inferred from sequencing data of *P. lambertiana* transcripts codifying for DCL proteins. (.pdf, 297 KB)
- Figure S13 - Secondary structure from three *P. lambertiana* miRNA precursors. (.pdf, 2.93 MB)
- File S2 - An extended description of the gene expression analysis. (.pdf, 85 KB)
- Figure S1 - Rarefaction curves of all libraries for each sequencing technology as an estimation of library sequencing saturation. (.pdf, 2.93 MB)
- Figure S2 - Transcript length distribution of assembled transcripts where no CDS was identified. (.pdf, 2.93 MB)
- Figure S3 - Box plots of transcript length distribution for different technologies and for ?embryo?, ?2 cm female cones? and ?female cones at time of pollination? samples. (.pdf, 2.93 MB)
- Figure S4 - Contribution of each technology to improve the coverage of the single mapping units (SMU) when it performed as the best one. (.pdf, 2.93 MB)
- Figure S5 - Number of splice variants provided by each technology in ?embryo?, ?2 cm female cones? and ?female cones at the time of pollination? samples. (.pdf, 165 KB)
- Figure S6 - Plant species with the most protein sequence similarity to *P. lambertiana* transcripts. (.pdf, 88 KB)
- Figure S7 - Transcriptome characterization by tissue samples. (.pdf, 141 KB)
- Table S1 - Tissue sample description and sequencing statistics. (.xls, 12 KB)
- Tables S2 - Number of raw transcripts (before transcript selection) with similarity to ribosomal RNA. (.xls, 9 KB)
- Table S3 - Splice variant counting in female cones (S and V) and embryo (E) libraries. (.xls, 6 KB)
- Table S4 - Statistically significant Gene Ontology (GO) terms identified in differentially expressed transcript sets. (.xls, 461 KB)
- Table S5 - Number of proteins that compose each conifer-specific protein families and protein domains identified in the family. (.xls, 39 KB)
- Table S6 - Functional annotation of sugar pine transcripts present in conifer-specific families identified by MCL analysis. (.xls, 184 KB)
- Table S7 - Number of proteins that compose each *P. lambertiana*-specific protein families and protein domains identified in the family. (.xls, 11 KB)
- Table S8 - Functional annotation of sugar pine transcripts present in *P. lambertiana*-specific families identified by MCL analysis. (.xls, 42 KB)
- Table S9 - MicroRNA precursors identified in *P. lambertiana* transcripts. (.xls, 23 KB)
