# Supplementary material for: Assessing the Gene Content of the Megagenome: Sugar Pine (Pinus lambertiana)
Source: G3 (Bethesda). 2016 Oct 31;6(12):3787–802. doi: 10.1534/g3.116.032805 (PMC5144951; doi:10.1534/g3.116.032805)
Supplement: Supplemental Material [file supp_g3.116.032805_FileS1.pdf]

Assessing the gene content of the megagenome : sugar pine (*Pinus lambertiana*)

Daniel Gonzalez-Ibeas[*,1], Pedro J. Martínez-García[†,1], Randi A. Famula[†], Annette Delfino-Mix[‡], Kristian A. Stevens[§], Carol A. Loopstra[**], Charles H. Langley[§], David B. Neale[†,2], Jill L. Wegrzyn[*,2]

[*]Department of Ecology and Evolutionary Biology, University of Connecticut, Storrs, CT USA
[†]Department of Plant Sciences, University of California, Davis, CA USA
[‡]USDA Forest Service, Institute of Forest Genetics, Placerville, CA, USA
[§]Department of Evolution and Ecology, University of California, Davis, CA USA
[**]Department of Ecosystem Science and Management, Texas A&M University, College Station, TX USA

[1]Equal contribution
[2]Corresponding authors

SUPPLEMENTAL TEXT

File S1

Female cones yielded a total of 6,543 SMUs (950 mapped in more than two locations and 1,639 were not mapped) of which 3,729 were mapped by HiSeq transcripts and 4,101 by PacBio, and cones samples (V) yielded 6,562 SMUs (885 mapped in more than two locations and 3,461 were not mapped), 3,860 and 4,099 were mapped by HiSeq and PacBio transcripts. A total of 1,196 and 1,397 were covered by both technologies, in both samples, respectively, that were used for the comparison. In female cones, for 1,129 SMUs HiSeq provided the longest hit compared to 217 by PacBio (150 SMUs were equivalent), so for 979 SMUs HiSeq performed as the best technology and for 67 was PacBio. After removal of outliers, the number of splice variants per SMU was 1.4 and 3.9 for HiSeq and PacBio, respectively. The same situation was observed for female cones (V) samples. For 1,294 SMUs HiSeq provided the longest hit compared to 416 by PacBio (313 SMUs were equivalent), so for 981 SMUs HiSeq performed as the best technology and for 103 was PacBio. After removal of outliers, the number of splice variants per SMU was 1.5 and 4.0 for HiSeq and PacBio, respectively (Figure S5). Figure for transcript length distribution and transcript coverage improvement are provided in Supplemental material (Figures S3 and S4).
