# Supplementary material for: Assessing the Gene Content of the Megagenome: Sugar Pine (Pinus lambertiana)
Source: G3 (Bethesda). 2016 Oct 31;6(12):3787–802. doi: 10.1534/g3.116.032805 (PMC5144951; doi:10.1534/g3.116.032805)
Supplement: Supplemental Material [file supp_g3.116.032805_FigureS8.pdf]

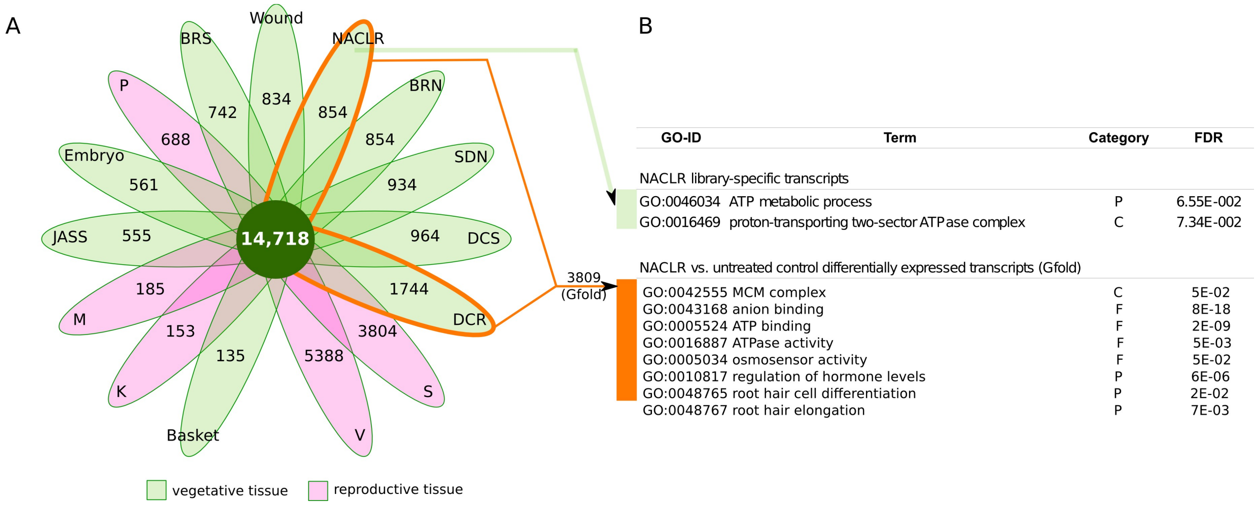

Figure S8. Treatment-specific *P. lambertiana* transcripts analysis. (A) Number of treatment-specific transcripts for each analyzed tissue (green background): K (pollen), M (early female cones (2 weeks) before pollination), P (pollen cones), E1 (seed), Basket (root), stem and needles), SDN (needle), BRS (stem), WS (stem), NACLR (root), JAS (stem), S (2 cm female cones), V (female cones at time of pollination), DCR (root), DCS (stem), BRN (needle). (B) Gene Ontology (GO) terms overrepresented in the library-specific NACLR transcripts (green), and differentially expressed transcripts in NaCl treated samples (NACLR) compared to the root healthy control (DCR) (orange), identified with Gfold.
