# Supplementary material for: Assessing the Gene Content of the Megagenome: Sugar Pine (Pinus lambertiana)
Source: G3 (Bethesda). 2016 Oct 31;6(12):3787–802. doi: 10.1534/g3.116.032805 (PMC5144951; doi:10.1534/g3.116.032805)
Supplement: Supplemental Material [file supp_g3.116.032805_FigureS9.pdf]

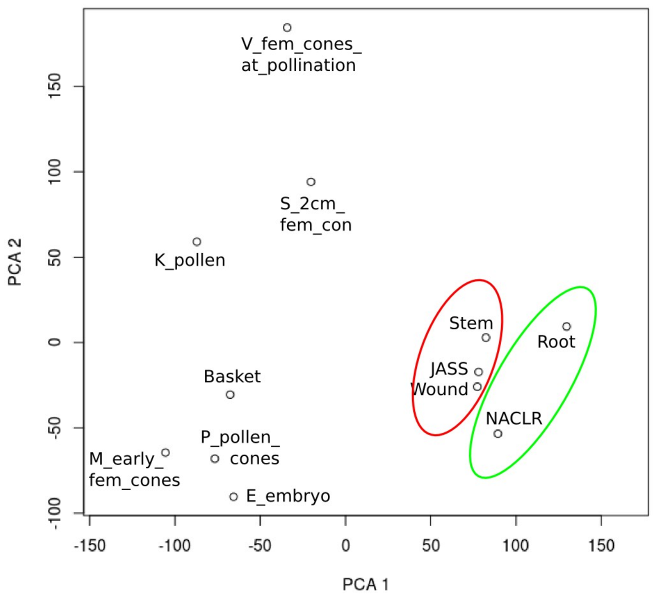

Figure S9. Principal component analysis (PCA) of sugar pine samples used for gene expression estimation. Red circle groups stem samples and green circle root samples. Reproductive and vegetative tissue samples grouped in opposite sides of the plot, except for Basket samples, we assumed due to be a mix of plant tissues. Transcriptomic changes were of higher magnitude for different developmental stages than for vegetative tissue after treatments (jasmonate, wounding or NaCl). It is also worth noting the roughly parallel direction of both components describing stem and root treatments.
