# Supplementary material for: Assessing the Gene Content of the Megagenome: Sugar Pine (Pinus lambertiana)
Source: G3 (Bethesda). 2016 Oct 31;6(12):3787–802. doi: 10.1534/g3.116.032805 (PMC5144951; doi:10.1534/g3.116.032805)
Supplement: Supplemental Material [file supp_g3.116.032805_FigureS10.pdf]

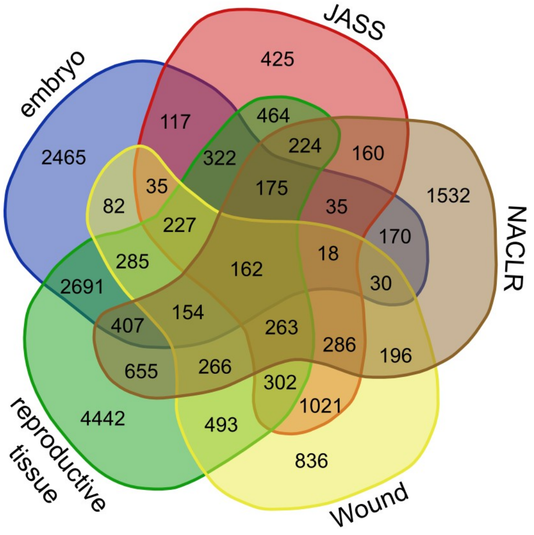

Figure S10. Number of unique and shared differentially expressed transcripts for stressed tissues, embryo samples and pooled reproductive tissues (cones and pollen).
