# Supplementary material for: Assessing the Gene Content of the Megagenome: Sugar Pine (Pinus lambertiana)
Source: G3 (Bethesda). 2016 Oct 31;6(12):3787–802. doi: 10.1534/g3.116.032805 (PMC5144951; doi:10.1534/g3.116.032805)
Supplement: Supplemental Material [file supp_g3.116.032805_FigureS11.pdf]

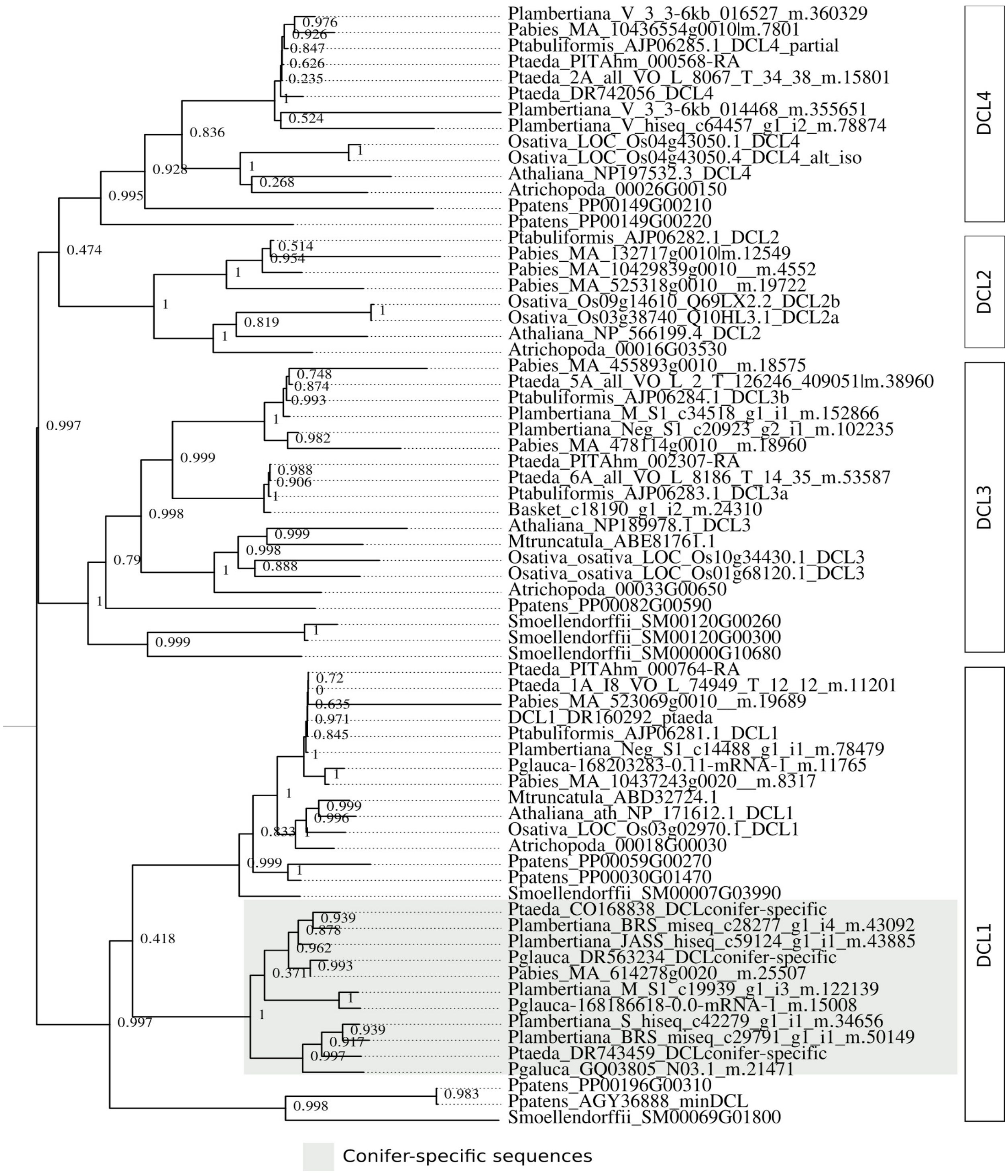

Figure S11. Phylogenetic analysis of DCL proteins from *P. lambertiana* and several plant species, including three conifers (*Pinus taeda (*Ptaeda*), Picea abies (*Pabies*), Picea glauca (*Pglauca*)* and *Pinus tabuliformis (*Ptabuliformis*)*), a monocot (*Oryza sativa (*Osativa*)*), a dicot (*Arabidopsis thaliana* (Athaliana), *Amborella trichopoda* (*Atrichopoda*), *Physcomitrella patens* (Ppatens) and *Selaginella moellendorffii* (Smoellendorffii). All DCL proteins grouped according the main four described DCLtypes. DCL1 clade was split in three subgroups, one corresponding to conventional plant DCL1s, other potentially being conifer-specific DCL1s (grey shaded), and the third one corresponding to truncated DCL1 variants of ancient plants.
