# Supplementary material for: Assessing the Gene Content of the Megagenome: Sugar Pine (Pinus lambertiana)
Source: G3 (Bethesda). 2016 Oct 31;6(12):3787–802. doi: 10.1534/g3.116.032805 (PMC5144951; doi:10.1534/g3.116.032805)
Supplement: Supplemental Material [file supp_g3.116.032805_FigureS12.pdf]

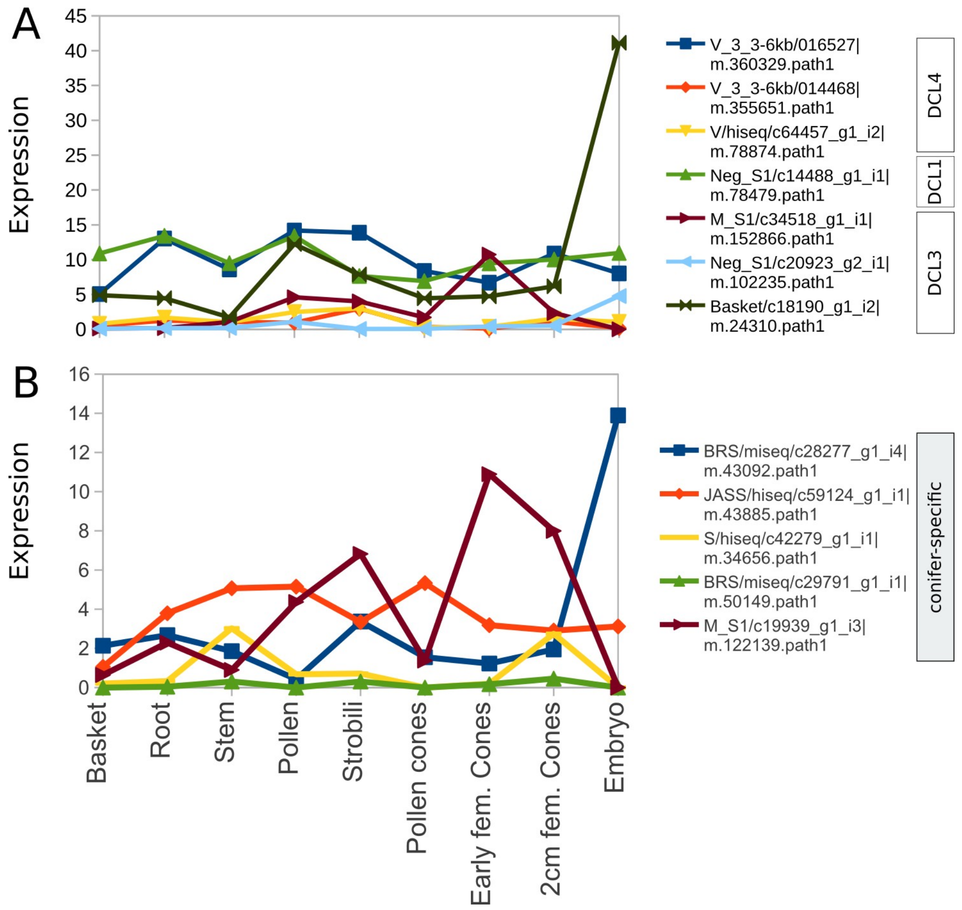

Figure S12. Gene expression analysis inferred from sequencing data of *P. lambertiana* transcripts codifying for DCL proteins. (A) Conventional DCL sequences with sequence similarity to other plant species. (B) Potential conifer-specific DCL1 proteins deducted from phylogenetic analysis (Figure S11-SN6).
