# Supplementary material for: Assessing the Gene Content of the Megagenome: Sugar Pine (Pinus lambertiana)
Source: G3 (Bethesda). 2016 Oct 31;6(12):3787–802. doi: 10.1534/g3.116.032805 (PMC5144951; doi:10.1534/g3.116.032805)
Supplement: Supplemental Material [file supp_g3.116.032805_FigureS13.pdf]

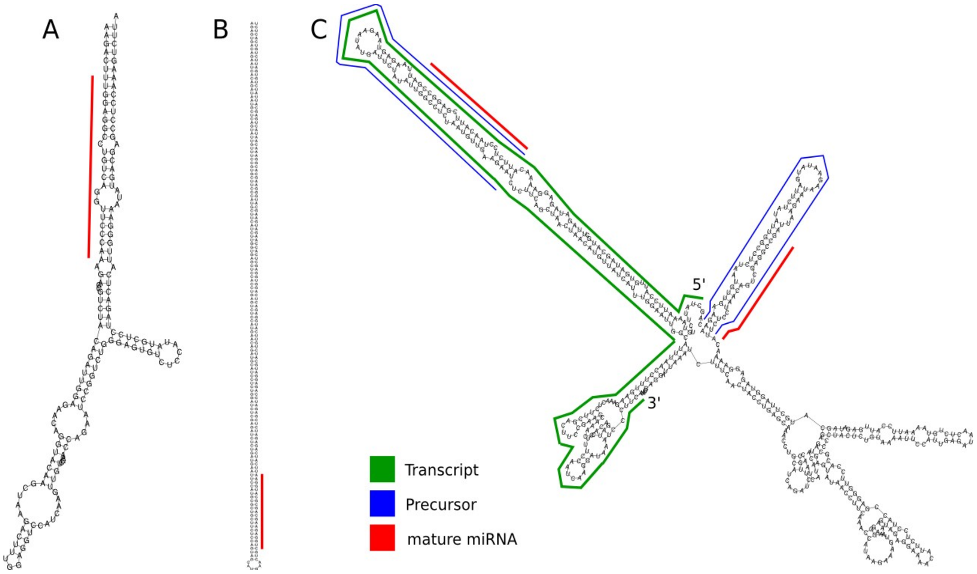

Figure S13. Secondary structure from three *P. lambertiana* miRNA precursors. (A) Precursor that meets canonical miRNA requirements. (B) Long precursor with low MFEI index resembling a fold-back transposon. (C) Secondary structure of genomic region potentially coding for two clustered miRNAs.
