# Supplementary material for: Assessing the Gene Content of the Megagenome: Sugar Pine (Pinus lambertiana)
Source: G3 (Bethesda). 2016 Oct 31;6(12):3787–802. doi: 10.1534/g3.116.032805 (PMC5144951; doi:10.1534/g3.116.032805)
Supplement: Supplemental Material [file supp_g3.116.032805_FileS2.pdf]

Assessing the gene content of the megagenome : sugar pine (*Pinus lambertiana*)

Daniel Gonzalez-Ibeas[*,1], Pedro J. Martínez-García[†,1], Randi A. Famula[†], Annette Delfino-Mix[‡], Kristian A. Stevens[§], Carol A. Loopstra[**], Charles H. Langley[§], David B. Neale[†,2], Jill L. Wegrzyn[*,2]

[*]Department of Ecology and Evolutionary Biology, University of Connecticut, Storrs, CT USA
[†]Department of Plant Sciences, University of California, Davis, CA USA
[‡]USDA Forest Service, Institute of Forest Genetics, Placerville, CA, USA
[§]Department of Evolution and Ecology, University of California, Davis, CA USA
[**]Department of Ecosystem Science and Management, Texas A&M University, College Station, TX USA

[1]Equal contribution
[2]Corresponding authors

SUPPLEMENTAL TEXT

File S2

Tissue and Library Characterization: NACLR sample (root after NaCl treatment) was used as a case study to compare library-specific (854) and differentially expressed (3,809) transcripts after treatment. A total of 854 transcripts were identified as library-specific. Gene Ontology (GO) term analysis of the library-specific set shows enrichment of categories potentially related to the Na+/H+ exchange by means of ATP hydrolysis for Na+ exclusion and/or vacuolar accumulation during the salt stress response (Figure S8). This is likely due to the presence of 11 transcripts annotated as ATPases, including three P-type ATPases (IPR008250, IPR006068, IPR004014) and two V-type ATPases (IPR002379). GO term analysis of the differentially expressed transcripts in treated samples relative to untreated control showed also categories related to ATPase and osmosensor activity, ion binding and root hair elongation. Among the NACLR-specific transcripts, only 233 were differentially expressed after treatment, including two of the P-type ATPases.

Transcript Abundance Estimation: Treated samples have been compared to their respective untreated control (see methods), and reproductive tissue has been compared to the basket stage seedling sample, as a mix of vegetative (needle, root and stem) tissue. On average, 5,958 transcripts were identified as differentially expressed in each sample with a fold change > 2.0. Gene Ontology terms overrepresented in differentially expressed genes were also assessed (Table S4, Table 3). A total of 3,809 differentially expressed transcripts were identified in NaCl treated

roots, with GO terms associated with genes involved in stress response, including DnaJ-like chaperone proteins, transcripts related to the glutathione and ubiquitin pathways, calcium-transporting ATPases, and ethylene-responsive transcription factors. Abiotic stresses, such as drought, salt and freezing, lead to the disruption of the plant water status that, in turn, provokes similar physiological consequences. We identified as differentially expressed several "early responsive to dehydration" transcripts. In *Arabidopsis*, these genes are rapidly induced by dehydration and encompass varied functions and localizations (Alves et al, 2011). For example, differentially expressed *P. lambertiana* transcripts in treated samples had sequence similarity to ERD1, ERD2 and ERD4 (a chloroplast ATP-dependent protease, a cytosolic HSP70 and a membrane protein). Genes related to the MCM complex were also differentially expressed. These were primarily helicases which are often up-regulated in response to salt stress in plants (Dang *et al.* 2011a, Dang *et al.* 2011b). These findings support studies that DNA replication machinery can be exploited for promoting stress tolerance in crops (Tuteja *et al.* 2012).

Genes related to osmosensor activity were uniquely over-represented in NaCl-treated samples, including three different histidine kinases, one similar to a two component release system. Several sensory histidine kinases have been reported to have a role as osmosensors in plants, indicating a possible cross-talk between hormone and stress responsive cascades (Nongpiur *et al.* 2012). Interestingly, this *P. lambertiana* transcript was down-regulated, in agreement with the role of some histidine kinases as negative regulators (Nongpiur *et al.* 2012; Tran *et al.* 2007). Other categories found in the NaCl treated samples were hormone signaling, including auxin and gibberellin which are expected reactions under salinity stress (Nongpiur *et al.* 2012; Singh, Singla-Pareek, and Pareek 2011). In addition, several genes related to root morphogenesis and root hair elongation, primarily cellulose synthase proteins, likely due to known modifications in cell wall structure, were enriched (Le Gall *et al.* 2015). Analysis of the library-specific (down/up)-regulated transcripts found a number of differentially expressed pentatricopeptide repeat proteins (PPR), which have been associated with abiotic stress responses for both organelle- and non-organeller-localized types. Overexpression of PPR proteins have been reported to increase salt tolerance in *Arabidopsis* (Barkan and Small 2014; Zsigmond *et al.* 2012; Jiang *et al.* 2015; Yuan and Liu 2012).

Jasmonic acid, a fatty-acid-derived signaling molecule, is involved in several aspects of plant biology including pollen and seed development, and defense against wounding, ozone, insect pests and microbial pathogens (Kunkel and Brooks 2002). In particular, methyl jasmonate is associated with increased resistance to fungal infection in Norway spruce (Krokene, Nagy, and Solheim 2008). Several differentially expressed genes flagged with the GO term "defense response" were identified in methyl-jasmonate-treated samples. These included 16 transcripts annotated as TIR-NBS-LRR class disease resistance proteins, a subfamily of NBS-LRR proteins involved in the detection of diverse pathogens, including bacteria, viruses, fungi, nematodes, insects and oomycetes (McHale *et al.* 2006). Additional genes included defensin-like, ethylene-responsive, heat shock proteins, WRKY transcription factors, and MYB transcription factors.

However, even though research on fatty acid-based signaling systems in plants has focused mainly on jasmonic acid, growing evidence suggests that compounds of the same or related biochemical pathways may have a role in signaling of pathogen defense, for example Cyclopentenones (Howe *et al* 2001). These compounds are oxylipids derived from polyunsaturated fatty acids and structurally similar to jasmonic acid. A few of these, such as 12-oxo-phytodienoic acid, are known to be a physiological signal for defense (Howe 2001). Fifteen genes were associated with "response to cyclopentenone" in methyl-jasmonate-treated samples. The majority of these were topoisomerases, likely participating in DNA re-modeling for transcription, but were surprisingly down-regulated. Several genes related to the gibberellin pathway were also differentially expressed. It has been described that plants promote defense over growth under pathogen signaling by interfering with gibberellin signaling cascade by means of jasmonate (Yang *et al.* 2012). The identification of these genes in treated samples may be reflecting this pathway cross-talk.

The comparison of reproductive tissues with vegetative revealed overwhelming overrepresentation of GO terms related to chloroplast localization, photosynthesis and thylakoid membrane. As expected, this was observed in all cone samples (male or female) and pollen. Other distinguishing features of reproductive tissue included a large number of differentially expressed MADS-box-like transcripts and squamosa-like transcription factors, known to participate in developmental processes in plants, including conifers (Gramzow and Theissen 2010; Mouradov *et al.* 1998). In the case of pollen samples, they were represented in categories such as "meristematic phase transition" or "pattern specification process". Gibberellic acid, a well-known plant hormone stimulating plant growth and development, is a tetracyclic di-terpenoid compound synthesized via the terpenoid pathway, and requires several types of enzymes, including terpene synthases (Gupta and Chakrabarty 2013). Down-regulation of several terpene synthases were responsible for the term "terpene synthase activity" in pollen samples. The most abundant protein domains identified in cone and pollen differentially expressed transcripts was "2OG-FeII_Oxy" (IPR005123), corresponding to 2–oxoglutarate-dependent dioxygenases (2OGDs), which are involved in a wide range of biological processes, including DNA demethylation, proline hydroxylation, plant hormone biosynthesis, and biosynthesis of gibberellins and flavonoids (Kawai, Ono, and Mizutani 2014).

Among the enriched processes in female cones, signal transduction (kinases and transmembrane proteins), transcripts related to the gibberellin pathway and several terms related to developmental processes, primarily squamosa and MADS-box-like transcripts, were identified. One transcript annotated with the protein domain Floricaula/leafy, was present in various plant development proteins that are homologous to Floricaula (FLO) and leafy (LFY) proteins (floral meristem identity proteins). Specific isoforms are known to be differentially expressed among male and female cones in *Pinus caribaea* (Dornelas and Rodriguez 2005). The same transcript in *P. lambertiana* was not differentially expressed in male cones, down-regulated in early female

cones, and over-expressed in 2 cm female cone samples, suggesting potential functional similarities. Several GO terms related to salicylic acid biosynthesis and metabolism were associated with differentially expressed genes. For example, this is seen in down-regulated genes of early female cone samples and up- and down-regulated genes in embryo samples. Generally studied because of its role in plant defense, it is worth noting that salicylic acid also has roles in plant development and seed germination (Vicente and Plasencia 2011).

REFERENCES (only supplemental text)

Alves, M. S., P. B. Reis, S.P. Dadalto, J. A. Q. A. Faria, E. P. B. Fontes *et al.*, 2011 A novel transcription factor, ERD15 (Early Responsive to Dehydration 15), connects endoplasmic reticulum stress with an osmotic stress-induced cell death signal. J. Biol. Chem. 286: 20020-20030.

Dang, H. Q., N. Q. Tran, R. Tuteja, and N. Tuteja, 2011a Promoter of a salinity and cold stress-Induced MCM6 DNA helicase from pea. Plant Signal. Behav. 6(7): 1006-8.

Dang, H. Q., N. Q. Tran, S. S. Gill, R. Tuteja, and N. Tuteja, 2011b A single subunit MCM6 from pea promotes salinity stress tolerance without affecting yield. Plant Mol. Biol. 76: 19-34.

Tuteja, N., S. S. Gill, A. F. Tiburcio, and R. Tuteja, 2012 *Improving crop resistance to abiotic stress*. Wiley-VCH Verlag GmbH & Co. KGaA, Weinheim, Germany.

Nongpiur, R., P. Soni, R. Karan, S. L. Singla-Pareek, and A. Pareek, 2012 Histidine kinases in plants. Plant Signal Behav. 7: 1230-37.

Tran, L. S. P., T. Urao, F. Qin, K. Maruyama, T. Kakimoto *et al.*, 2007 Functional analysis of AHK1/ATHK1 and cytokinin receptor histidine kinases in response to abscisic acid, drought, and salt stress in *Arabidopsis*. Proc. Natl. Acad. Sci. U. S. A. 104: 20623-20628.

Singh, K., S. L. Singla-Pareek, and A. Pareek, 2011 Dissecting out the crosstalk between salinity and hormones in roots of *Arabidopsis*. OMICS 15: 913-924.

Le Gall, H., F. Philippe, J. M. Domon, F. Gillet, J. Pelloux *et al.*, 2015 Cell wall metabolism in response to abiotic stress. Plants 4(1): 112-66.

Barkan, A., and I. Small, 2014 Pentatricopeptide repeat proteins in plants. Annu. Rev. Plant Biol. 65: 415-42.

Zsigmond, L., A. Szepesi, I. Tari, G. Rigo, A. Kiraly *et al.*, 2012 Overexpression of the mitochondrial PPR40 gene improves salt tolerance in *Arabidopsis*. Plant Sci. 182: 87-93.

Jiang, S. C., C. Mei, S. Liang, Y. T. Yu, K. Lu *et al.*, 2015 Crucial roles of the pentatricopeptide repeat protein SOAR1 in *Arabidopsis* response to drought, salt and cold stresses**.** Plant Mol. Biol. 88: 369-85.

Yuan, H., and D. Liu, 2012 Functional disruption of the pentatricopeptide protein SLG1 affects mitochondrial RNA editing, plant development, and responses to abiotic stresses in *Arabidopsis*. Plant J. 70: 432-444.

Kunkel, B. N., and D. M. Brooks, 2002 Cross talk between signaling pathways in pathogen defense. Curr. Opin. Plant Biol. 5: 325-331.

Krokene, P., N. E. Nagy, and H. Solheim, 2008 Methyl jasmonate and oxalic acid treatment of Norway spruce: anatomically based defense responses and increased resistance against fungal infection. Tree Physiol. 28: 29-35.

McHale, L., X. Tan, P. Koehl, and R. W. Michelmore, 2006 Plant NBS-LRR proteins: adaptable guards. Genome Biol. 7(4): 212.

Howe, G. A., 2001 Cyclopentenone signals for plant defense: Remodeling the jasmonic acid response. Proc. Natl. Acad. Sci. U. S. A. 98 (22): 12317-19.

Yang, D. L., J. Yao, C. S. Mei, X. H. Tong, L. J. Zeng *et al.*, 2012 Plant hormone jasmonate prioritizes defense over growth by interfering with gibberellin signaling cascade. Proc. Natl. Acad. Sci. U. S. A. 109: E1192-E1200.

Gramzow, L., and G. Theissen, 2010 A hitchhiker's guide to the MADS world of plants. Genome Biol. 11(6): 214

Gupta, R., and S. K. Chakrabarty, 2013 Giberellic acid in plant. Plant Signal. Behav. 8 (9).

Kawai, Y., E. Ono, and M. Mizutani, 2014 Evolution and diversity of the 2–oxoglutarate-dependent dioxygenase superfamily in plants. Plant J. 78: 328-43.

Dornelas, M. C., and A. P. M. Rodriguez, 2005 A FLORICAULA/LEAFY gene homolog is preferentially expressed in developing female cones of the tropical pine Pinus caribaea var. caribaea. Genet. Mol. Biol. 28: 299-307.

Vicente, M. R. S., and J. Plasencia, 2011 Salicylic acid beyond defence: its role in plant growth and development. J. Exp. Bot. 62: 3321-3338.
