# Supplementary material for: Assessing the Gene Content of the Megagenome: Sugar Pine (Pinus lambertiana)
Source: G3 (Bethesda). 2016 Oct 31;6(12):3787–802. doi: 10.1534/g3.116.032805 (PMC5144951; doi:10.1534/g3.116.032805)
Supplement: Supplemental Material [file supp_g3.116.032805_FigureS1.pdf]

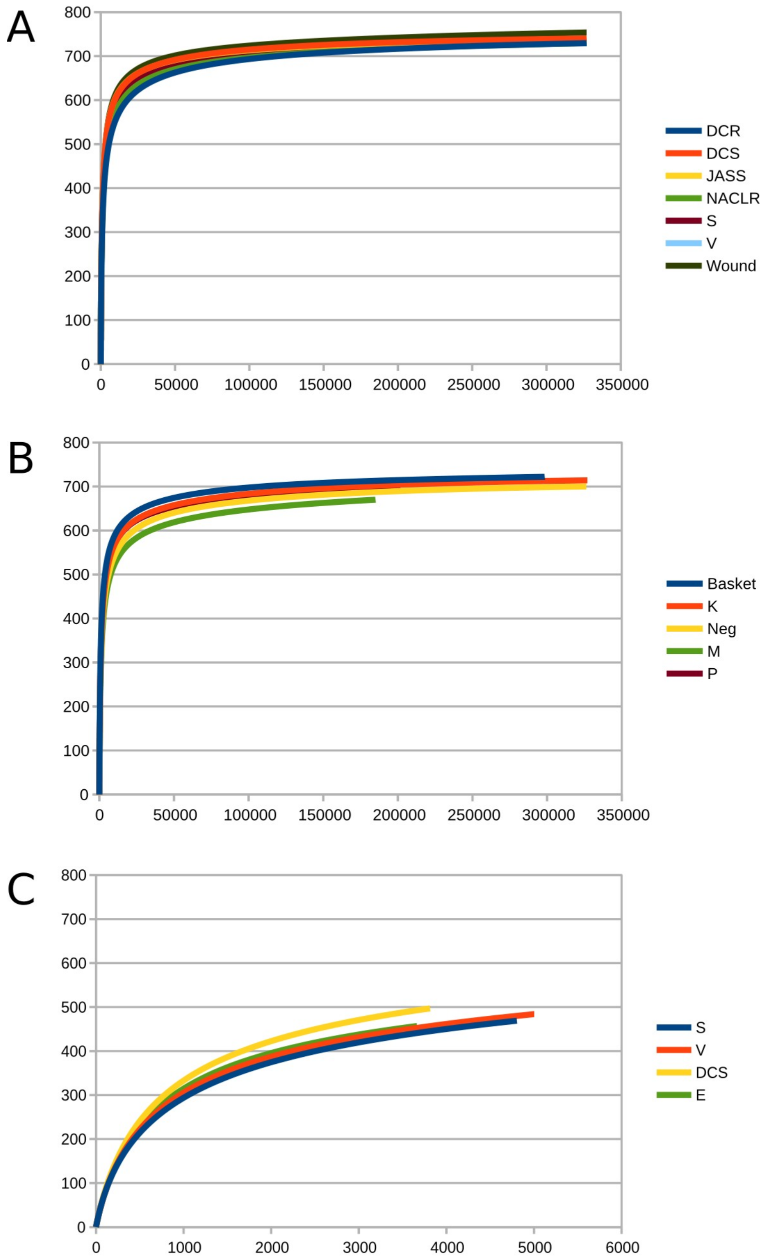

Figure S1. Rarefaction curves of all libraries for each sequencing technology as an estimation of library sequencing saturation. (A) HiSeq (B) MISeq (C) PacBio.
