# Supplementary material for: Assessing the Gene Content of the Megagenome: Sugar Pine (Pinus lambertiana)
Source: G3 (Bethesda). 2016 Oct 31;6(12):3787–802. doi: 10.1534/g3.116.032805 (PMC5144951; doi:10.1534/g3.116.032805)
Supplement: Supplemental Material [file supp_g3.116.032805_FigureS2.pdf]

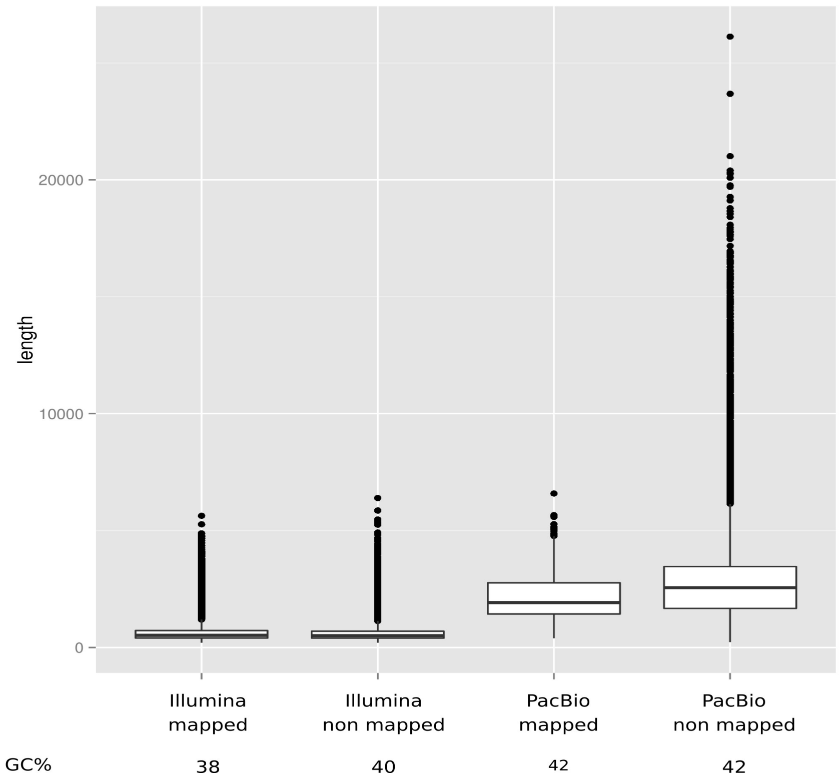

Figure S2. Transcript length distribution of assembled transcripts where no CDS was identified. Data are shown for Illumina or PacBio libraries, and for transcripts that could be aligned (or not aligned) on the *P. lambertiana* genome. Average GC content of transcripts is shown in the bottom of the figure.
