# Supplementary material for: Assessing the Gene Content of the Megagenome: Sugar Pine (Pinus lambertiana)
Source: G3 (Bethesda). 2016 Oct 31;6(12):3787–802. doi: 10.1534/g3.116.032805 (PMC5144951; doi:10.1534/g3.116.032805)
Supplement: Supplemental Material [file supp_g3.116.032805_FigureS3.pdf]

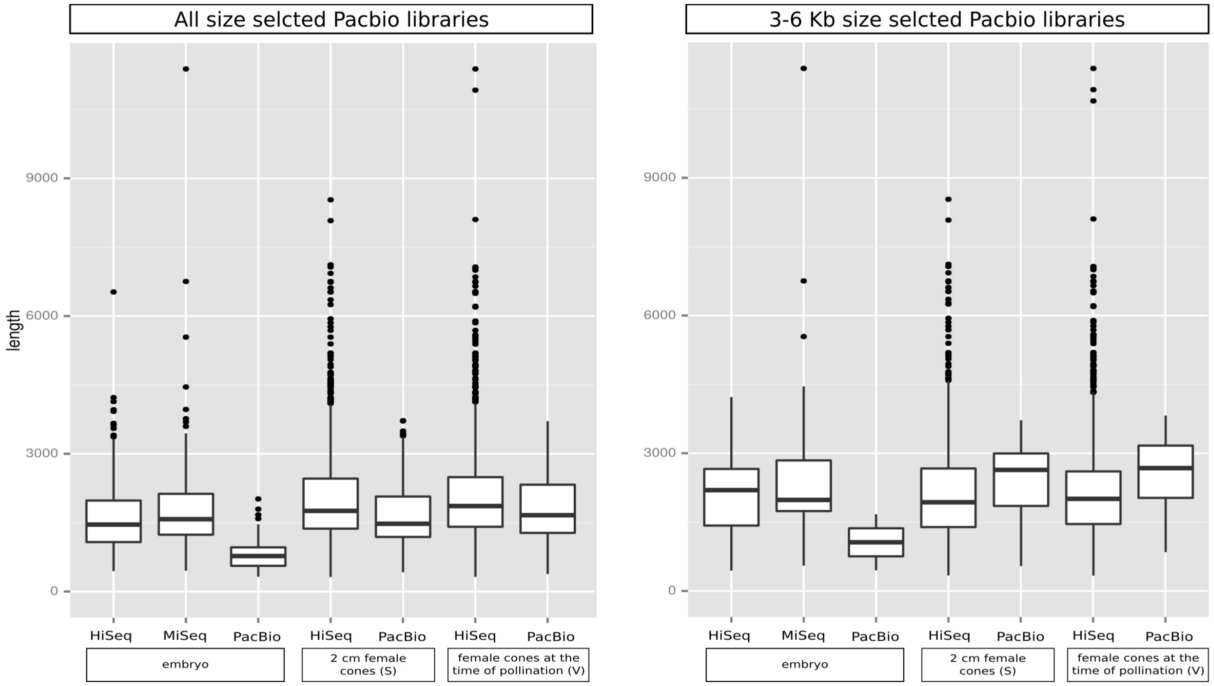

Figure S3. Box plots of transcript length distribution for different technologies and for "embryo", "2 cm female cones" and "female cones at time of pollination" samples. Data are presented by using all size selected PacBio libraries (left) and only 3-6 Kb size selected PacBio libraries (right) in the comparisons.
