# Supplementary material for: Assessing the Gene Content of the Megagenome: Sugar Pine (Pinus lambertiana)
Source: G3 (Bethesda). 2016 Oct 31;6(12):3787–802. doi: 10.1534/g3.116.032805 (PMC5144951; doi:10.1534/g3.116.032805)
Supplement: Supplemental Material [file supp_g3.116.032805_FigureS4.pdf]

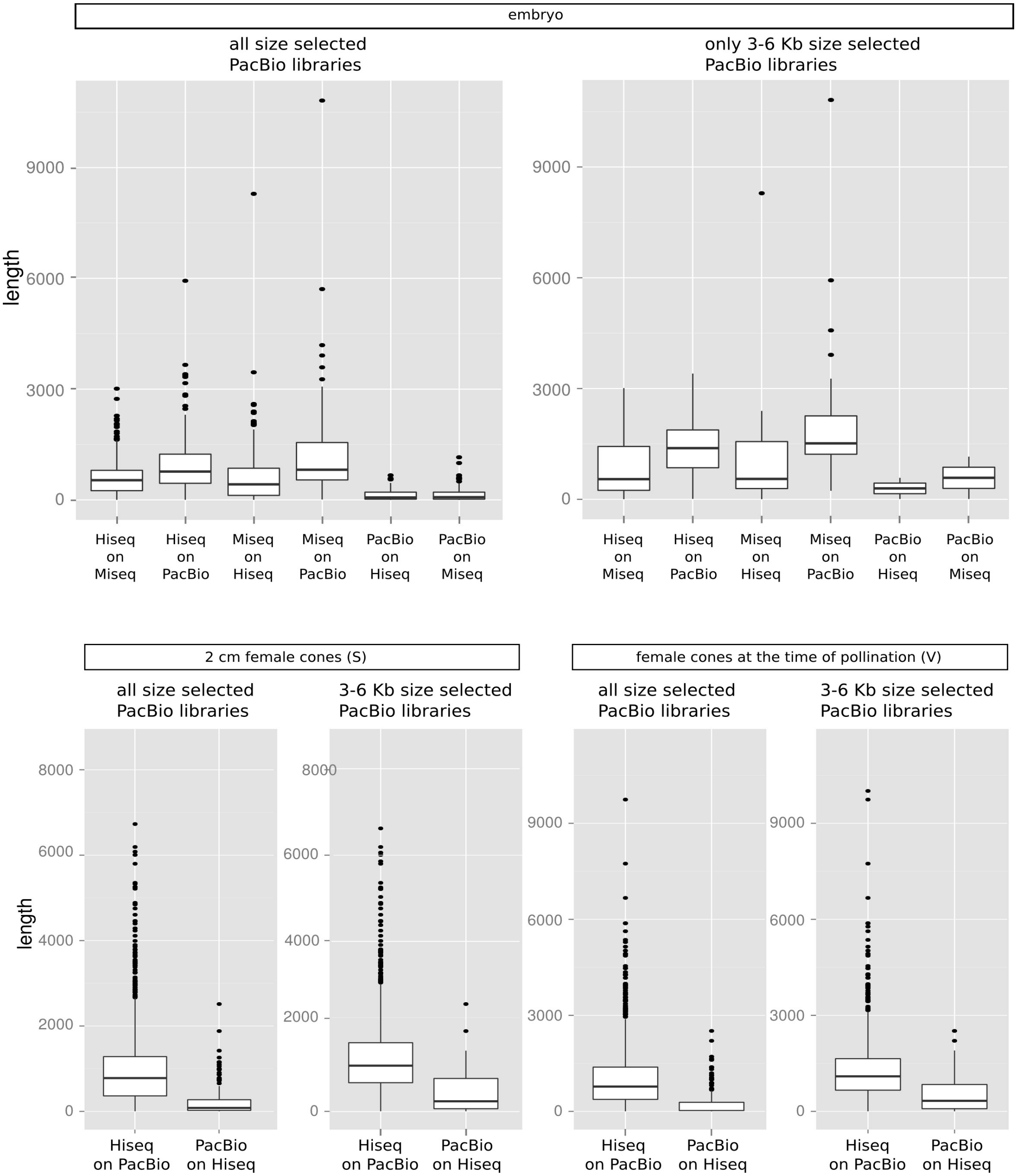

Figure S4. Contribution of each technology to improve the coverage of the single mapping units (SMU) when it performed as the best one. Data are presented for “embryo” (top, 3 technologies; HiSeq, MiSeq and PacBio), “2 cm female cones” (bottom left, 2 technologies: HiSeq and PacBio) and “female cones at time of pollination” (bottom right, 2 technologies: HiSeq and PacBio) samples. For each sample, data are shown by using all size selected or only 3-6 Kb size selected Pacbio libraries.
