# Supplementary material for: Assessing the Gene Content of the Megagenome: Sugar Pine (Pinus lambertiana)
Source: G3 (Bethesda). 2016 Oct 31;6(12):3787–802. doi: 10.1534/g3.116.032805 (PMC5144951; doi:10.1534/g3.116.032805)
Supplement: Supplemental Material [file supp_g3.116.032805_FigureS7.pdf]

A

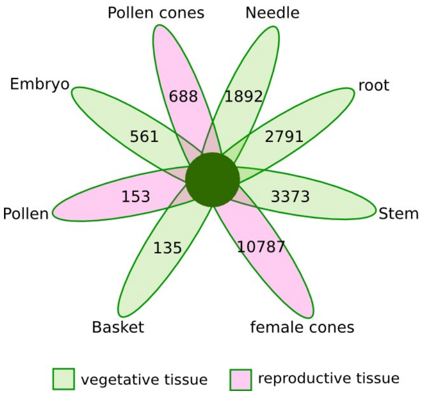

B

Stem	Root	Needle	Basket	Embryo	Reproductive tissue
5270	3312	2612	337	--	--
4711	3190	2455	--	1218	--
3932	2936	2102	--	--	7133

Figure S7. Transcriptome characterization by tissue samples. (A) number of identified tissue-specific transcripts. (B) Number of unique transcripts when comparing the three vegetative tissues (stem, root and needle) with basket, embryo and pooled reproductive tissues (cones and pollen).
